# A genome-wide atlas of human cell morphology

**DOI:** 10.1038/s41592-024-02537-7

**Published:** 2025-01-27

**Authors:** Meraj Ramezani, Erin Weisbart, Julia Bauman, Avtar Singh, John Yong, Maria Lozada, Gregory P. Way, Sanam L. Kavari, Celeste Diaz, Eddy Leardini, Gunjan Jetley, Jenlu Pagnotta, Marzieh Haghighi, Thiago M. Batista, Joaquín Pérez-Schindler, Melina Claussnitzer, Shantanu Singh, Beth A. Cimini, Paul C. Blainey, Anne E. Carpenter, Calvin H. Jan, James T. Neal

**Affiliations:** 1 Broad Institute of MIT and Harvard, Cambridge, MA USA; 2 Type 2 Diabetes Systems Genomics Initiative, Broad Institute of MIT and Harvard, Cambridge, MA USA; 3 Calico Life Sciences LLC, South San Francisco, CA USA; 4 The Novo Nordisk Foundation Center for Genomic Mechanisms of Disease, Broad Institute, Cambridge, MA USA; 5 Diabetes Unit and Center for Genomic Medicine, Massachusetts General Hospital, Boston, MA USA; 6 Department of Biological Engineering, MIT, Cambridge, MA USA; 7 Koch Institute for Integrative Research, MIT, Cambridge, MA USA; 8 Present Address: Stanford University, Stanford, CA USA; 9 Present Address: Genentech Department of Cellular and Tissue Genomics, South San Francisco, CA USA; 10 Present Address: University of Pennsylvania, Philadelphia, PA USA

**Keywords:** High-throughput screening, Fluorescence imaging, Software, Image processing, High-throughput screening

## Abstract

A key challenge of the modern genomics era is developing empirical data-driven representations of gene function. Here we present the first unbiased morphology-based genome-wide perturbation atlas in human cells, containing three genome-wide genotype–phenotype maps comprising CRISPR–Cas9-based knockouts of >20,000 genes in >30 million cells. Our optical pooled cell profiling platform (PERISCOPE) combines a destainable high-dimensional phenotyping panel (based on Cell Painting) with optical sequencing of molecular barcodes and a scalable open-source analysis pipeline to facilitate massively parallel screening of pooled perturbation libraries. This perturbation atlas comprises high-dimensional phenotypic profiles of individual cells with sufficient resolution to cluster thousands of human genes, reconstruct known pathways and protein–protein interaction networks, interrogate subcellular processes and identify culture media-specific responses. Using this atlas, we identify the poorly characterized disease-associated TMEM251/LYSET as a Golgi-resident transmembrane protein essential for mannose-6-phosphate-dependent trafficking of lysosomal enzymes. In sum, this perturbation atlas and screening platform represents a rich and accessible resource for connecting genes to cellular functions at scale.

## Abstract (web summary)

An optical pooled cell profiling platform (PERISCOPE) based on Cell Painting and optical sequencing of molecular barcodes was used to develop the first unbiased genome-wide morphology-based perturbation atlas in human cells.

## Main

Large-scale DNA sequencing has transformed our ability to identify and catalog diverse genotypic information but created a new bottleneck: characterizing the diverse impacts of genotype on human biology. Thus, systematically connecting human genes and genotypes to disease- and trait-relevant phenotypes remains a grand challenge for biomedicine.

Pooled CRISPR screens^1^ have proven a powerful tool for tackling this challenge, but typically require compromising on either phenotypic content or scale. Genome-scale pooled CRISPR screens enable systematic assessment of gene function but compatible phenotypes, such as proliferation or cell death, are often simple or require a targeted assay, making them inappropriate for assessing many biologically relevant processes in human cells, which are often subtle, graded and/or complex^2^. In contrast, high-content profiling approaches such as imaging, transcriptomics, proteomics and metabolomics can capture hundreds of quantitative phenotypes for each sample, providing a rich phenotypic profile, but are typically incompatible with genome-scale perturbation. A notable exception is Perturb-seq^3–6^, which has very recently been applied to profile the effects of CRISPR interference (CRISPRi) knockdown (KD) of the expressed genome of the human chronic myeloid leukemia cell line K562 (ref. ^7^). This study demonstrated the immense value of generating rich, high-dimensional representations of cell state at genome scale using a new (and not yet widely available) DNA sequencing technology^8,9^ and resource-intensive data generation effort.

Optical pooled screening, which combines image-based phenotyping with image-based sequencing of perturbation barcodes, has emerged as a promising and complementary approach for high-dimensional genotype–phenotype mapping at single-cell resolution that is scalable and cost effective^10–13^. Optical pooled screens enable quantitative assessment of phenotypes invisible to molecular profiling approaches, such as cell morphology and subcellular localization, with greater throughput than arrayed image-based screens^14^ and, in contrast to pooled enrichment-based imaging approaches^15–18^, have no requirement for physical selection or predefinition of phenotypes.

Here, we combined unbiased high-dimensional image-based cell phenotyping with massively parallel optical pooled CRISPR screens to build the first genome-scale perturbation atlas of morphology phenotypes in human cells. We report the design of an optimized cell phenotyping panel based on the popular Cell Painting^19,20^ image-based profiling assay that enables five-color fluorescence microscopy of cell phenotypes followed by four-color in situ sequencing by synthesis (ISS) to assign perturbations to cells. We also built a scalable, open-source, cloud-based pipeline for generating barcoded image-based profiles from genome-scale perturbation datasets. We use this technology to execute two whole-genome pooled optical CRISPR screens in human cervical cancer cells (HeLa) cultured either in traditional cell culture medium or physiologic medium^21^, profiling the effects of >20,000 single gene knockouts (KOs) in unbiased fashion and mapping genome-wide gene-by-environment interactions. We additionally apply our approach in human lung cancer cells (A549). Together, this work establishes a valuable resource for connecting human genotypes to high-dimensional image-based cellular phenotypes at scale.

## Results

### High-dimensional optical CRISPR screens at genome scale

To assess genome-wide KO effects on cell morphology, we first constructed a whole-genome CRISPR guide RNA library optimized for optical screening. To build this library, we selected, on average, four single guide (sg)RNAs per gene from existing libraries^22,23^, identifying sgRNA sequences that would allow for total deconvolution of the sgRNA library in 12 cycles of ISS while also allowing for a Levenshtein distance of 2 between sgRNA sequences to enable error detection^24^, resulting in a library containing 80,408 sgRNAs targeting 20,393 genes (Supplementary Table 1). We cloned the sgRNA library into the CRISPR droplet sequencing (CROP-seq) vector^5^, enabling expression and direct ISS of sgRNA sequences (henceforth referred to as barcodes) and packaged it for lentiviral delivery.

**Fig. 1:**
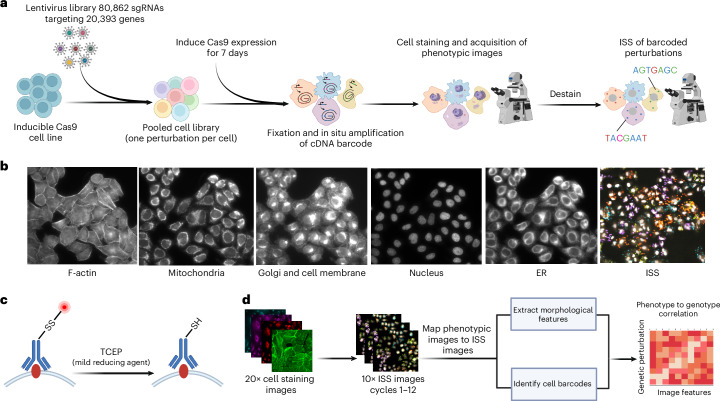
Pooled optical screens with PERISCOPE. **a**, The experimental workflow for PERISCOPE screens. **b**, Example images of five phenotypic stains and fluorescent ISS. **c**, A schematic of the destaining strategy to enable ISS after fluorescence imaging of phenotypic stains. SS, disulfide linked fluorophore; SH, reduced disulfide linkage. **d**, An overview of the PERISCOPE analysis pipeline including extraction of phenotypic features, deconvolution of barcodes and genotype–phenotype correlation. Figure created with BioRender.com.

**Extended Data Fig. 1:**
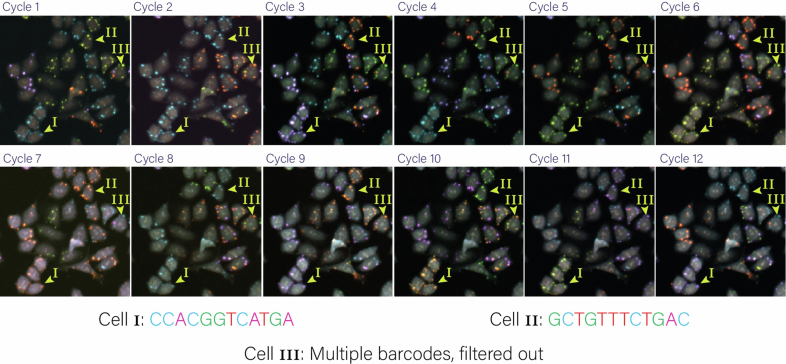
Example barcode calling based on twelve in-situ cycles. An example of a group of cells tracked over the twelve cycles of in-situ sequencing to call barcodes. Cells I and II highlight how the signal from fluorescent nucleotides are translated into a barcode read over twelve cycles.

To comprehensively map genome-wide gene KO effects to high-dimensional image-based phenotypes, we built a high-throughput data generation and analysis pipeline, perturbation effect readout in situ with single-cell optical phenotyping (PERISCOPE), comprising a suite of highly scalable wet and dry laboratory protocols that enables facile screening of genome-scale perturbation libraries by optical profiling. We first developed an optimized, destainable variant of the Cell Painting panel to collect morphological data by fluorescence imaging of cell compartments, followed by ISS of sgRNAs to assign perturbations to cells (Fig. 1a). This approach results in five phenotypic images for each cell—phalloidin (actin), anti-TOMM20 antibody (mitochondria), wheat germ agglutinin (WGA) (Golgi and cell membrane), concanavalin A (ConA) endoplasmic reticulum (ER)) and 4,6-diamidino-2-phenylindole (DAPI) (nucleus)—plus 12 sequencing images, which are used to identify sequential sgRNA bases (Fig. 1b and Extended Data Fig. 1). To overcome spectral overlap between fluorescent phenotyping markers and fluorescent sequencing signal, we conjugated phenotypic probes to fluorophores using a disulfide linker^25,26^. This strategy allows five-color labeling followed by treatment with tris(2-carboxyethyl)phosphine (TCEP), a reducing agent, resulting in linker cleavage and liberation of linked fluorophores, freeing up fluorescent channels for ISS (Fig. 1c). To analyze these data, we modified the standard Cell Painting image analysis workflow within the open source image analysis software CellProfiler^27^ to handle the added complexity of pooled perturbations, including the incorporation of image alignment across different resolutions and barcode calling^12^ (Fig. 1d). Similarly, we adapted our data analysis workflow based on the open-source Pycytominer^28^ library (Methods) to process single-cell profiles using pooled data rather than arrayed data.

### Morphology-based genome-wide perturbation maps in HeLa cells

**Extended Data Fig. 2:**
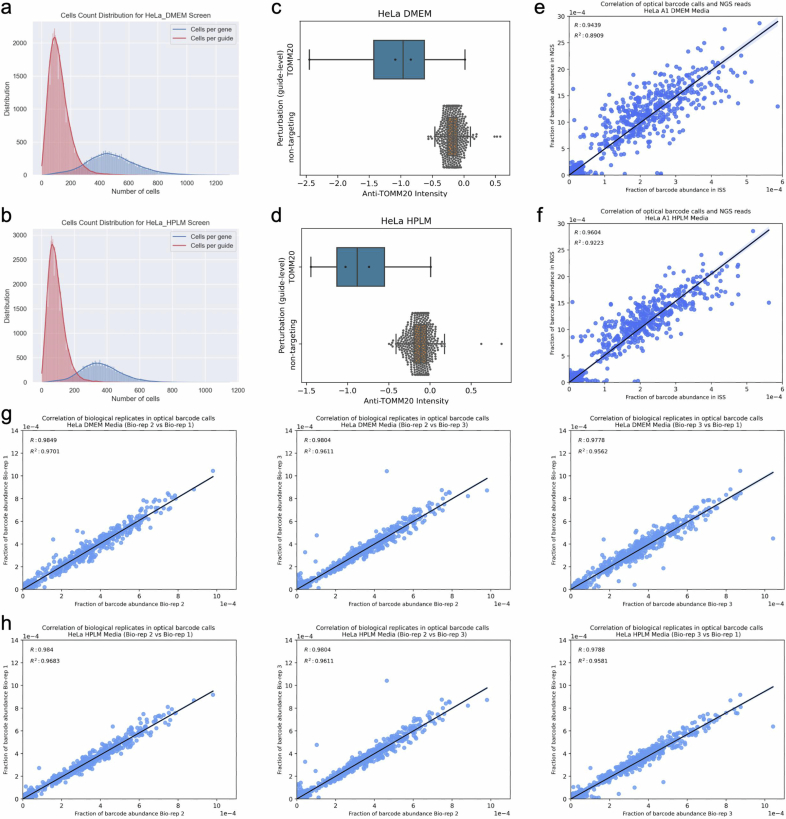
Technical summary of the HeLa whole genome screens. The distribution for the number of cells per gene and per guide present in the HeLa DMEM (**a**) and HPLM (**b**) dataset. (**c-d**) The distribution of normalized mean intensity in the mitochondrial channel from guide aggregated profiles in HeLa DMEM (**c**) and HeLa HPLM (**d**). Every dot overlaid on the boxplots represents a sgRNA (n=4 for guides targeting the TOMM20 gene and n=450 for nontargeting guides). The boxplots display the data as a distribution where the box spans from the first to the third quartile with the median as the center line. The whiskers extend to the maximum range of the distribution within 1.5 times the interquartile range. (**e-f**) Comparison of the relative abundance of sgRNA barcodes as quantified by NGS or in situ sequencing in HeLa DMEM (R^2^ = 0.89) (**e**) and HeLa HPLM (R^2^ = 0.92) (**f**), n= 75,000. Comparison of the relative abundance of barcodes as quantified by in situ sequencing among 3 different biological replicates representing individual viral transductions in HeLa DMEM (R_1to2_^2^ = 0.97, R_1to3_^2^ = 0.95, R_2to3_^2^ = 0.96) (**g**) and HeLa HPLM (R_1to2_^2^ = 0.97, R_1to3_^2^ = 0.96, R_2to3_^2^ = 0.96) (**h**), n=84,000. The correlation coefficients in (**e-h**) are calculated using Pearson correlation, and the solid black line represents a linear regression fit of the data, with the shaded region around the regression line indicating the 95% confidence interval calculated using the standard error of the regression.

We first aimed to demonstrate the scalability and robustness of the PERISCOPE pipeline by executing two whole-genome pooled optical CRISPR screens in human cervical cancer cells (HeLa) in separate growth media (Dulbecco’s modified Eagle medium (DMEM) and human plasma-like medium (HPLM), detailed below). For the HeLa DMEM screen, we used 30 identically prepared wells of six-well plates and collected morphological profiles from 12,312,520 individual cells yielding 20,421 gene-level profiles with an average 491 cells per gene (s.d. of 655) and 125 cells per guide (s.d. of 327) (cell coverage numbers exclude nontargeting controls, which are overrepresented; Extended Data Fig. 2a). Similarly, the HeLa HPLM screen was 24 wells, 9,111,690 cells, 20,420 gene-level profiles, 366 cells per gene (s.d. of 364) and 93 cells per guide (s.d. of 181). As expected, the PERISCOPE pipeline reported that perturbation of TOMM20, the direct target of the antibody stain for mitochondria, impacted the expected mitochondrial features (Extended Data Fig. 2c,d). Crucially, optical sgRNA counts were highly correlated with counts obtained from next-generation sequencing (NGS) of perturbed cells (Extended Data Fig. 2e,f), validating ISS accuracy and between biological screen replicates, confirming screen robustness (Extended Data Fig. 2g,h).

**Fig. 2:**
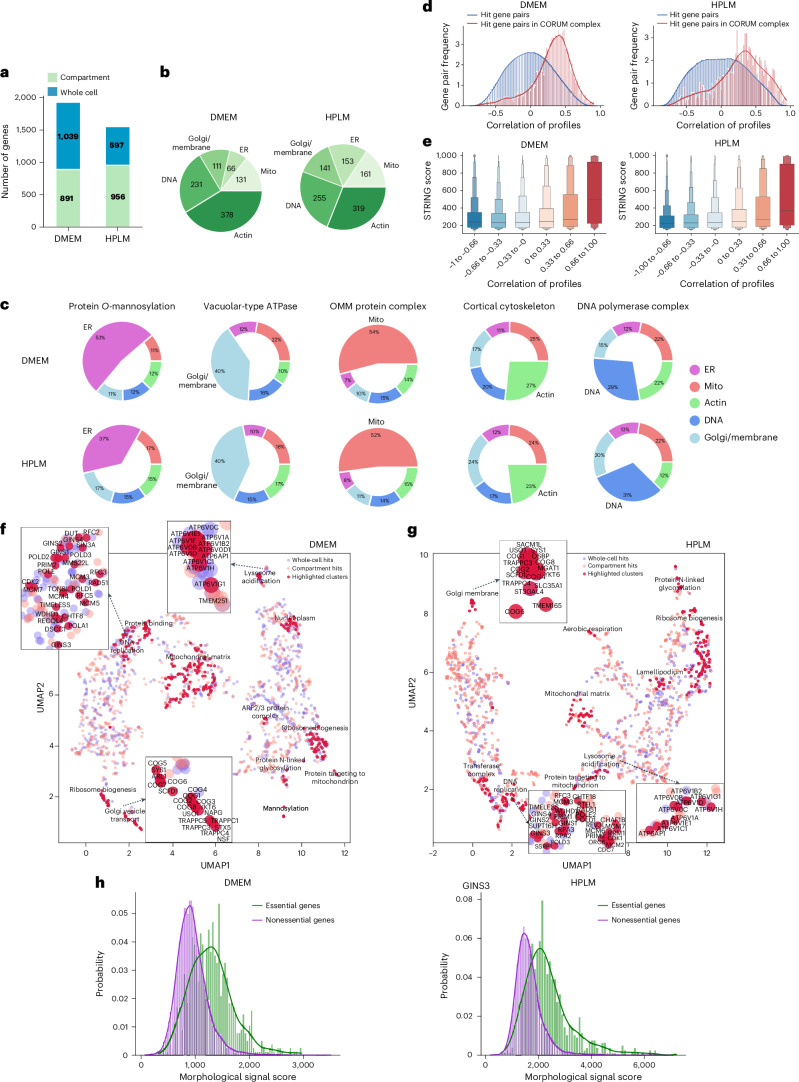
Summary of the results from two PERISCOPE screens at the whole-genome scale performed in HeLa cells in two growth media (DMEM and HPLM). **a**, A bar graph representing the number of hit genes identified. Green represents hit genes based on individual compartments (ER, mitochondria (mito), actin, DNA and Golgi/membrane) and blue represents hit genes based on the overall profile. **b**, The distribution of hit genes based on individual compartments (from **a**). It is possible for a gene to be hit in multiple compartments without being a whole-cell hit, see Extended Data Fig. 4c–f for details. **c**, Pie charts showing the average normalized fraction of the number of features significantly different from controls in each phenotypic channel for the genes in the indicated set. Filled wedges represent the channel in which the protein products are known to be present. **d**, The distributions of optical profile correlations between random hit gene pairs (blue) versus correlations between gene pairs in CORUM4.0 protein complexes (red). **e**, A boxen plot (letter-value plot) representing STRING scores divided into bins based on PERISCOPE profile correlation between gene pairs, *n* = 1,930 genes for DMEM and *n* = 1,553 genes for HPLM. The boxen plots display the data as a distribution where the center line represents the median, the central box represents the interquartile range from 25th to 75th percentile and the subsequent boxes represent increasingly narrower quantiles calculated for half of the remaining data. **f**,**g**, UMAP embedding of the hit gene profiles from the HeLa DMEM (**f**) or HPLM (**g**) dataset. Each dot represents a genetic perturbation and distance implies the correlation of profiles in a two-dimensional embedding. Manual annotation of cluster functions are presented for highlighted clusters based on GO datasets. Example insets show coherent clustering of related genes. **h**, The distribution of morphological signal scores for essential and nonessential genes (DepMap gene effect at −0.5 threshold) for all perturbations in the HeLa DMEM and HPLM datasets.

**Extended Data Fig. 3:**
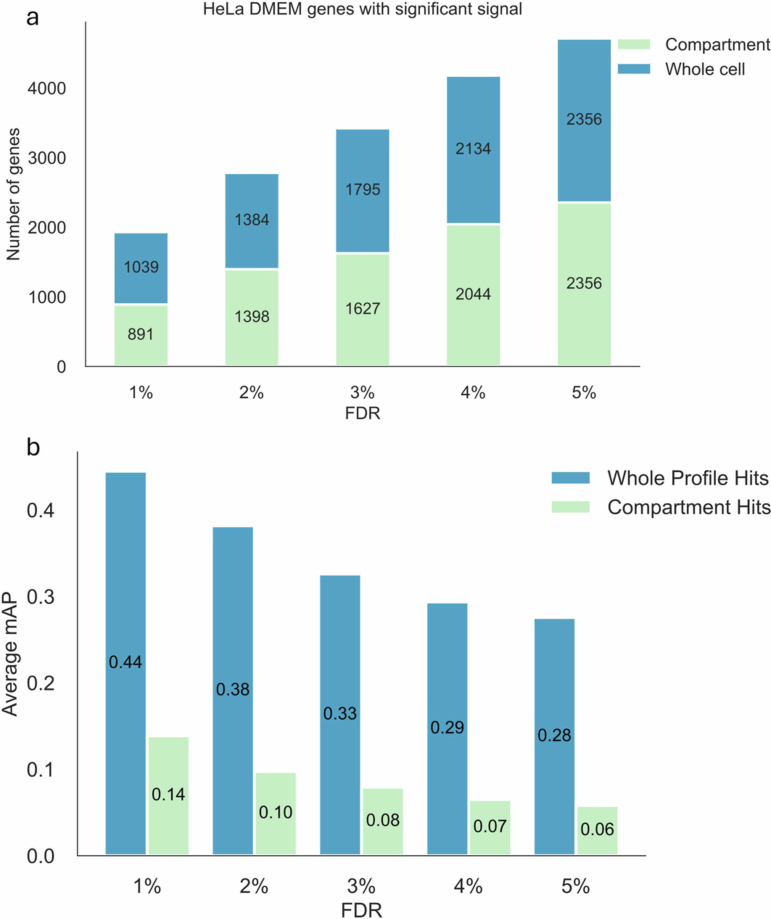
Number of hits and levels of guide similarity at different false discovery rates for the HeLa DMEM screen. (**a**) Bar graph of the number of hit genes identified in the HeLa DMEM screen at different false discovery rates. Green represents hit genes called based on single compartments (ER, Mitochondria, Actin, DNA and Golgi/Membrane) and blue represents hit genes called based on overall gene profile. Detailed description in the methods section. (**b**) Bar graph of the mean average precision (mAP) for hit perturbations in the HeLa DMEM screen at different false discovery rates. mAP was calculated by scoring each guide's ability to retrieve other guides targeting the same gene from the pool of all non-targeting guides based on cosine similarity and is a measure of phenotypic activity.

We next applied a hit calling pipeline that we designed to identify gene perturbation signatures above background noise using image-based features. Optical profiling collects spatial information and thus our pipeline was able to identify two classes of screen hit: ‘whole-cell’ hit genes, which were defined based upon aggregate signal from all cell compartments in a manner typical of image-based profiling experiments, and ‘compartment’ hit genes identified by imaging measurements from a subset of the five labeled subcellular compartments (Methods). Using a false discovery rate (FDR) of 1%, we identified 891/956 whole-cell hit genes, and 1,039/597 compartment hit genes, for a total of 1,930/1,553 hits (DMEM/HPLM) (Fig. 2a and Supplementary Table 2). As the choice of FDR cut-off is arbitrary, we show that less stringent FDRs produce larger hit lists (Extended Data Fig. 3a) with a corresponding decrease in average profile strength (Extended Data Fig. 3b) calculated using a metric to detect perturbation signal against a background comprising negative controls (mAP)^29^. Unsurprisingly, the whole-profile hits show much higher profile strength than compartment hits since the availability of complete profile information enhances signal detection.

**Extended Data Fig. 4:**
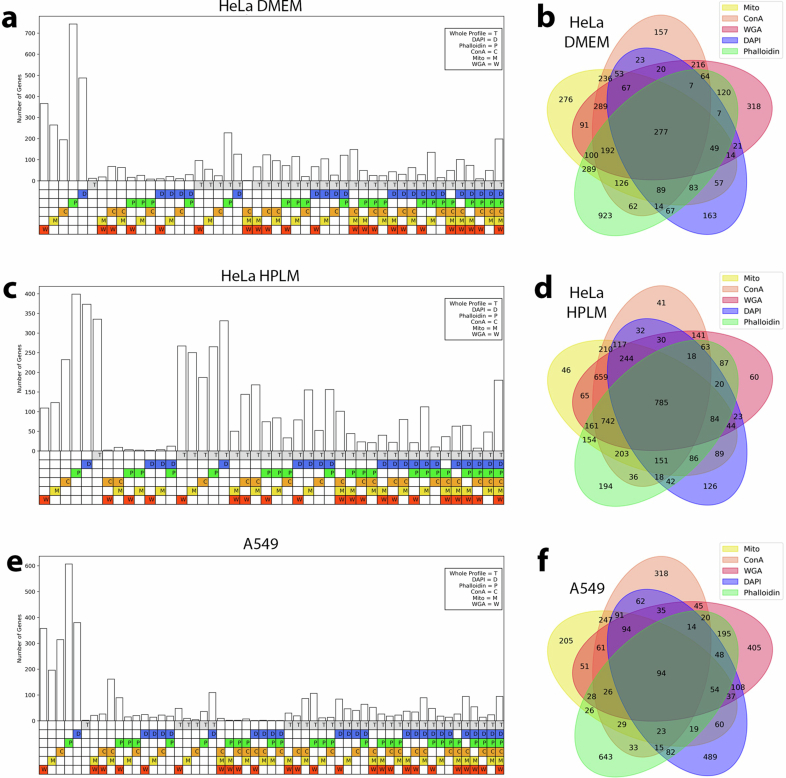
Hit genes can be called in multiple channel combinations. Genes called as hits in the HeLa DMEM (**a-b**), HeLa HPLM (**c-d**), and A549 (**e-f**) screens can be called as hits because of significant perturbation to their whole profile, any individual screen channel, or any combination thereof. Specific combinations without any hit genes are omitted from the bar plots (**a,c,e**) and whole profile hit information is omitted from the Venn diagrams (**b,d,f**) for clarity.

We next performed descriptive analyses of our hits to demonstrate biological signal in these screens. We found compartment hit genes in each subcellular compartment, demonstrating that each channel is providing useful information (Fig. 2b and Extended Data Fig. 4a–d). Importantly, we also observed that knocking out genes known to act in well-defined cell compartment-specific roles produced strong morphological phenotypes in those compartments. Specifically, we selected genes encoding five compartment-associated protein complex members and grouped their morphological profiles by complex. For each of these complexes, we observed an enrichment in phenotypic features extracted from the expected cellular compartment (Fig. 2c). For example, while perturbations targeting outer mitochondrial membrane proteins produce morphological phenotypes throughout the cell, a plurality (54% DMEM/52% HPLM) of the overall signal is concentrated in the mitochondria. Likewise, sgRNAs targeting genes involved in protein mannosylation display an enrichment in phenotypic features from the ER, where synthesis of mannosyl donor substrates and mannosyltransfer to proteins takes place^30^. Unsurprisingly, genes involved in highly pleiotropic processes (for example, cortical cytoskeleton), produce effects across cell compartments.

We next benchmarked image-based gene KO profiles against existing databases of gene function. First, using profile correlation between gene KOs as a proxy for functional similarity between genes, we compared our screen data with the protein–protein interaction databases CORUM^31^ and STRING^32^. Of 1,930/1,553 total hits, we identified 877/671 genes belonging to 1,350/953 unique complexes present in the CORUM4.0 database (DMEM/HPLM, respectively). Profiles from hit gene pairs within a cluster showed higher correlation values than the background distribution of all possible hit gene pairs (Fig. 2d). Additionally, morphological profile pairs with higher correlations demonstrated higher protein–protein interaction confidence scores from the STRING database (Fig. 2e).

**Extended Data Fig. 5:**
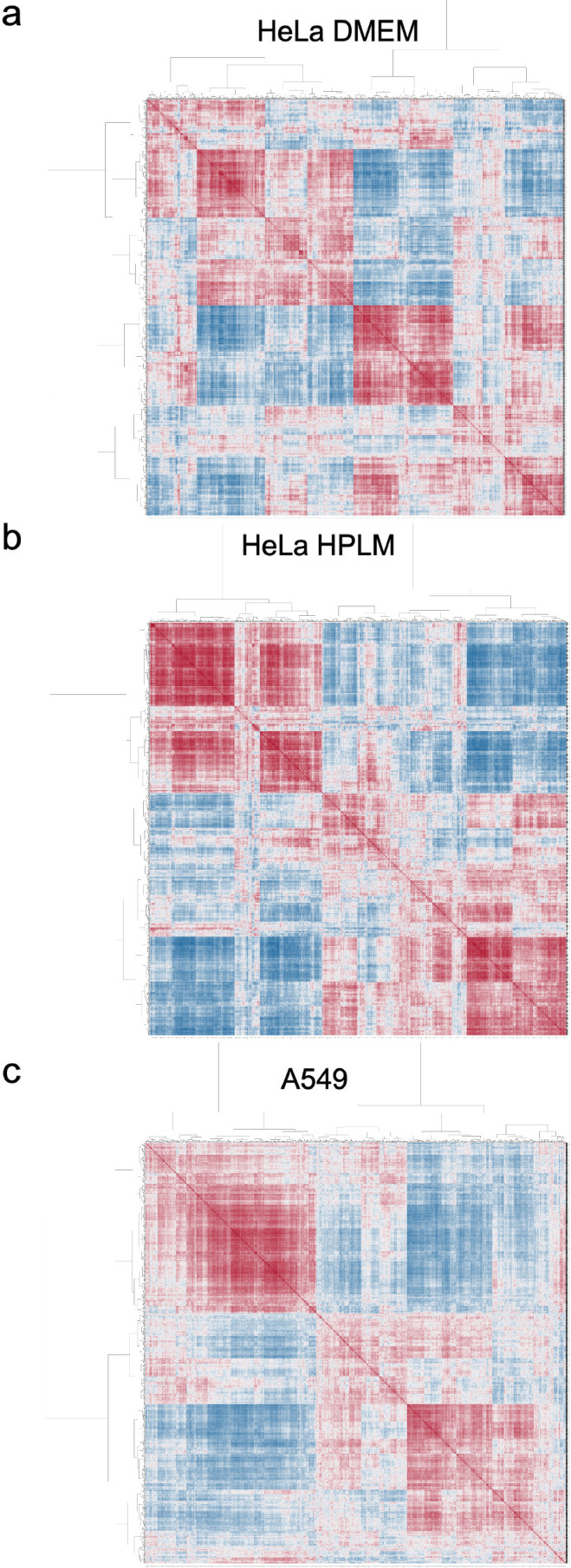
Clustering by optical profiles from all hit perturbations from whole genome screens. Heatmaps representing Pearson’s correlation between gene profiles after hierarchical clustering using Ward's method. The gene profiles come from the hit perturbations from HeLa DMEM (**a**), HeLa HPLM (**b**), and A549 (**c**) datasets. High resolution versions are available at https://github.com/broadinstitute/2022_PERISCOPE.

**Extended Data Fig. 6:**
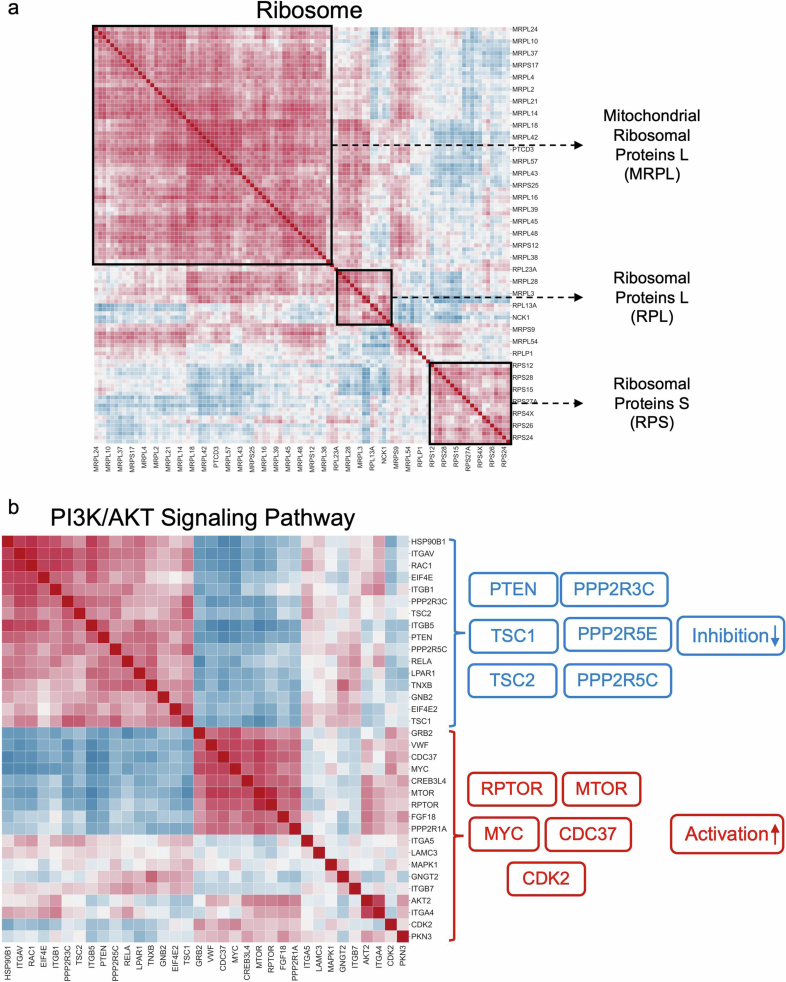
Hierarchical clustering of high dimensional morphological profiles captures physical interactions and signaling pathway relationships in HeLa DMEM data. (**a**) Ribosomal genes show enrichment in clusters that recapitulate known protein complexes as highlighted in the heatmap. Ribosome image created with Biorender. (**b**) The PI3K/AKT Signaling Pathway forms clusters where the correlation/anti-correlation in morphological profiles recapitulates the known activatory/inhibitory effects of genes, as annotated. Heatmaps are of Pearson’s correlation between gene profiles after hierarchical clustering using Ward's method.

We performed unbiased clustering of screen hits based on morphological similarity, and visualized high-level similarity between morphological profiles via two-dimensional uniform manifold approximation and projection (UMAP) embedding (Fig. 2f,g). We observed logical clustering by biological function across an array of processes, such as DNA replication, lysosome acidification, Golgi vesicle transport, messenger RNA processing, ribosome biogenesis, protein N-linked glycosylation, mannosylation, aerobic respiration and others. Hierarchical clustering of all hit genes based on the full high-dimensional profiles also revealed biologically coherent clustering of perturbations targeting related genes (Extended Data Fig. 5a,b). For example, targeted exploration of the hierarchical clusters in the DMEM condition shows that genes encoding various types of ribosomal proteins are largely grouped into three distinct clusters (Extended Data Fig. 6a). The largest cluster is enriched for genes encoding the large and the small subunits of the mitochondrial ribosome, which is essential in the translation of mitochondrial genes^33^, while two other clusters show enrichment for components of the large 60S subunit and the small 40S subunit of the mature 80S eukaryotic ribosome, respectively^34^. This example highlights the ability of optical pooled screens to capture structural information, as recently demonstrated^10^. We also found that signaling pathways were often well captured: as an example, perturbations targeting the phosphatidylinositol 3‑kinase/AKT serine–threonine protein kinase (PI3K/AKT) signaling pathway largely fall into two distinct clusters (Extended Data Fig. 6b). This pathway is involved in the cell cycle, growth and proliferation, and implicated in the progression of various cancers.^35^ Interestingly, components that have a stimulatory effect on the pathway such as RPTOR, MTOR or MYC strongly correlate with each other and also demonstrate significant anticorrelation to inhibitory factors such as PTEN, TSC1 or TSC2. The ability of the morphological profiles to distinguish the directionality of the signaling factors is a useful tool in understanding the underlying biology.

**Extended Data Fig. 7:**
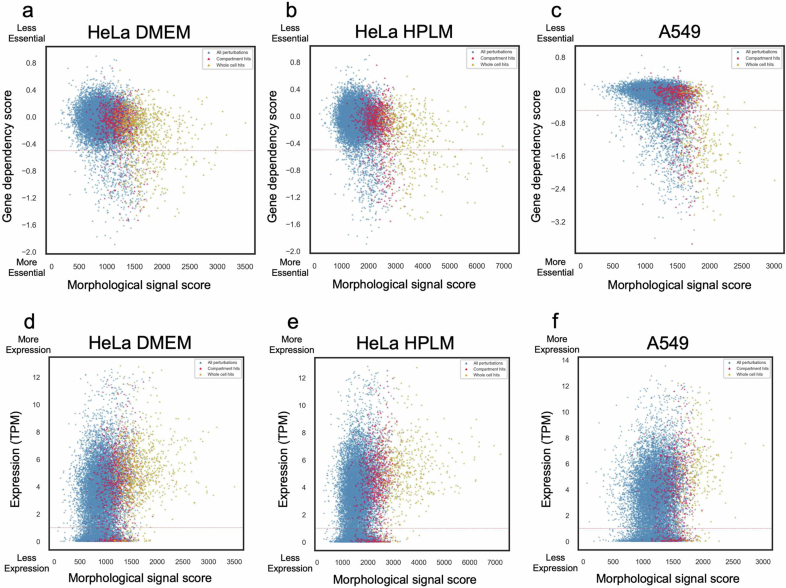
Morphological signal score is not well correlated with gene dependency or baseline gene expression. Comparison of the distribution of morphological signal scores and gene dependency scores for the HeLa DMEM (**a**), HeLa HPLM (**b**) and A549 (**c**) datasets. The gene dependency score was estimated using DEMETER2 for HeLa cells and DepMap for A549 cells. The dashed red line at −0.5 threshold highlights likely essential genes. Comparison of the distribution of morphological signal scores and gene expression TPM values for genes with TPM value > 0 for the A549 (**d**), the HeLa DMEM (**e**) or HeLa HPLM dataset (**f**). TPM values are inferred from RNA-seq data in DepMap data using the RSEM tool. In all panels, blue points indicate all perturbations, red points compartment hits, and yellow points whole cell hits.

We subsequently evaluated the extent to which image-based gene KO profiles were correlated with gene KO fitness effects using the Broad Institute’s Dependency Map (DepMap) database^36^. While essential genes were more likely, on average, to produce a high signal score (Methods), the majority of screen hits (80.4%/75.6% for DMEM/HPLM) were nonessential genes, consistent with most gene KOs producing optical phenotypes beyond simple cell toxicity (Fig. 2h and Extended Data Fig. 7a–c). Previous work has shown that Cell Painting can detect many specific cell health readouts including cell viability and cell cycle, so that even cytotoxic perturbations, such as KO of essential genes, can generate distinct morphological profiles^37^. Further, morphological signal score was not well correlated with baseline gene expression, as many genes expressed at low levels still produce significant morphological signal when perturbed (Extended Data Fig. 7d,e).

### Comparing gene-by-environment interactions at genome scale

Cell metabolism is influenced by a vast array of interactions between genes and environmental stimuli and, as such, in vitro genetic screens carried out in traditional cell culture media, which poorly recapitulate physiologic environments, may fail to capture metabolically relevant phenotypes. Recently, in contrast to typical laboratory culture medium DMEM, ‘physiologic media’ such as Plasmax^38^ or HPLM^39^ have been developed as tools to study the effects of genetic perturbations under environmental conditions designed to more accurately mimic in vivo human physiology and, in a recent study, HPLM was shown to dramatically alter the spectrum of gene essentiality in K562 cells^21^. Such studies demonstrate the usefulness of screening under physiologically relevant conditions, but have been limited to growth assays, preventing the systematic assessment of gene perturbation on high-dimensional cell phenotypes.

**Fig. 3:**
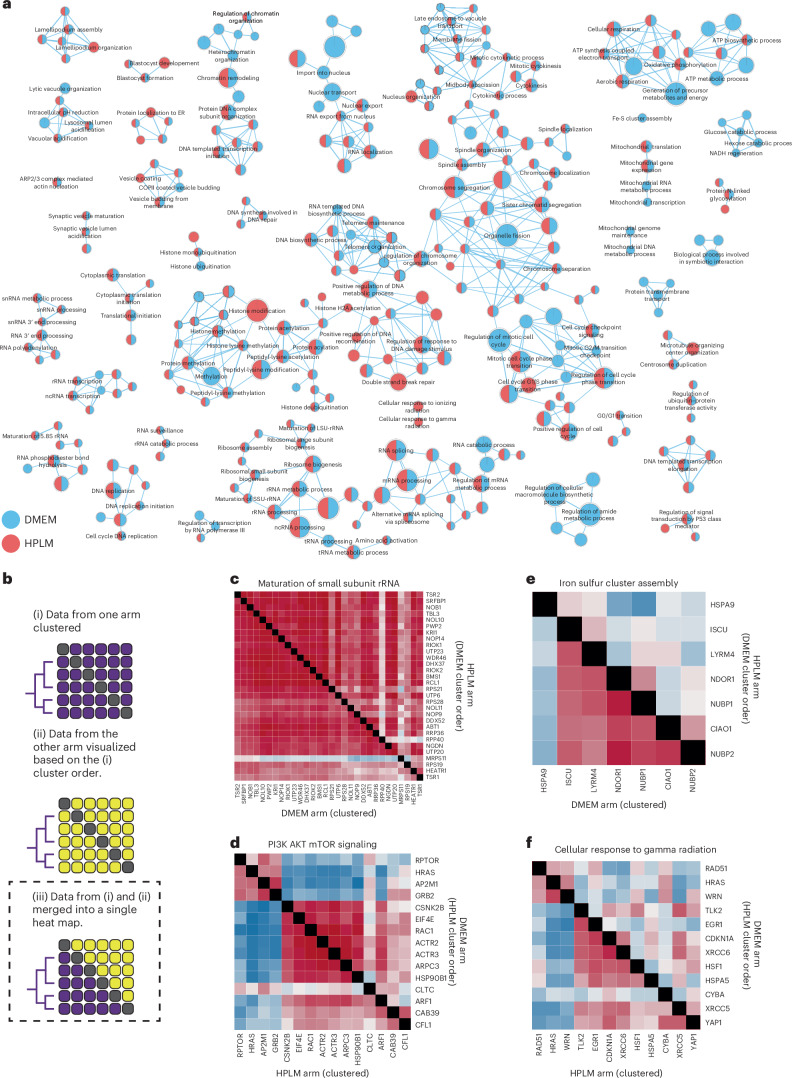
PERISCOPE identifies media-specific perturbation signatures. **a**, Enrichment map for biological processes based on profile signal strength between the HeLa DMEM and HPLM screens. The enrichment map was generated using a preranked GSEA analysis with a list of all genes ordered based on the calculated signal strength as described in methods. The GO: Biological Processes (GO:BP) gene set was employed for the enrichment analysis. Some of the labels and single/double nodes are not shown here for clarity. LSU rRNA, large subunit ribosomal RNA; snRNA, small nuclear RNA. **b**, A schematic for the generation of comparative diagonally merged heat maps. **c**–**f**, The heat maps display the Pearson’s correlation between gene profiles from both HeLa screens and are hierarchically clustered using Ward’s method on a single screen, with the sister screen plotted in the same order: we observed gene clusters enriched in both screens (for example, maturation of small subunit RNA (**c**) and PI3K AKT mTOR signaling (**d**)), as well as gene clusters enriched only in the DMEM condition (for example, the iron sulfur cluster assembly (**e**)) or HPLM condition (for example, the cellular response to gamma radiation (**f**)). The heat maps present hit genes in the GO:BP maturation of the small subunit ribosomal ribonucleic acid (SSU rRNA) gene set (GO:0030490) (**c**), hit genes in the hallmark PI3K AKT mTOR signaling gene set (**d**), hit genes in the GO:BP iron sulfur cluster assembly genes set (GO:0016226) (**e**) and hit genes in the GO:BP cellular response to gamma radiation (GO:0071480) (**f**).

In addition to their experimental tractability and prior validation in optical screening workflows^10–12^, HeLa cells have been demonstrated to exhibit sensitivity to metabolic environmental cues such as altered glucose levels^40,41^. To investigate these differences, we performed gene set enrichment analysis (GSEA) between the HeLa screens, a computational method that determines whether there are statistically significant differences between two biological states using ranked lists^42,43^. Our lists were ranked by the strength of each gene’s profile compared with control profiles using their ‘morphological signal score’ (Methods). On the basis of the GSEA analysis, 391 gene sets were enriched in the DMEM screen and 321 were enriched in the HPLM screen (Supplementary Table 3). Of these, 275 were common between the two screens, 116 were specific to the DMEM screen and 46 were specific to the HPLM screen. We visualized the GSEA results in a gene enrichment map (Fig. 3a).

To further visualize similarities between screens, we generated comparative diagonally merged heat maps, wherein only hits from both screens are plotted, the cluster order is set by one arm and the second arm is plotted in the same order (Fig. 3b–f). We observed that many genetic perturbations yield similar morphological impacts in both media types. For example, genes associated with small subunit ribosomal RNA maturation (Fig. 3c) and PI3K AKT mTOR signaling (Fig. 3d) exhibited strong similarity in pattern and strength of correlations in both DMEM and HPLM. These similarities in correlation patterns and strength across a variety of core processes in the same cell line indicate shared central biology and consistency of the screening method. We also observed that iron sulfur cluster assembly (Fig. 3e), which is required for mitochondrial respiration^44^, mitochondrial RNA metabolic processes and mitochondrial transcription processes was selectively enriched in the DMEM screen. Taken together, the overall enrichment of hits associated with central carbon metabolism in the DMEM screen may be reflective of metabolic differences induced by high (>25 mM) glucose levels present in DMEM^21^. Conversely, we also observed selective enrichment of processes in the HPLM screen related to DNA damage repair, such as cellular response to gamma radiation (Fig. 3f), positive regulation of DNA recombination and double-stranded break repair. This process enrichment is also probably linked to metabolic rewiring induced by substantial decreases in glucose and glutamine upon culture in HPLM as HeLa cells have been previously shown to exhibit hallmarks of DNA damage when cultured with reduced concentrations of these nutrients^45^.

### Morphology-based genome-wide perturbation maps in human lung cancer cells

**Fig. 4:**
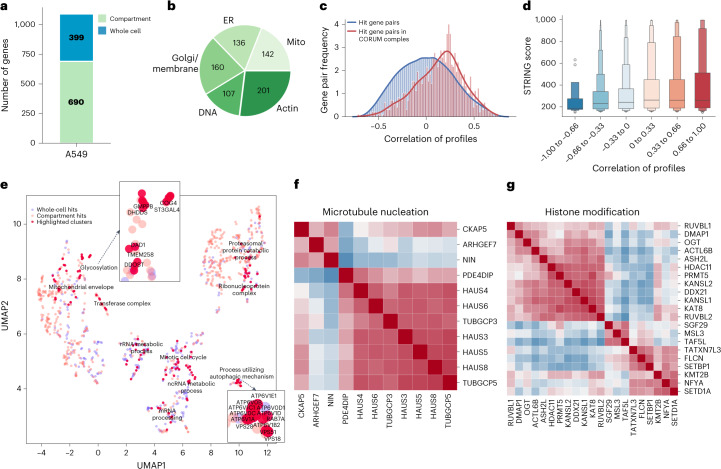
A genome-wide perturbation map in A549 cells. A summary of the whole-genome PERISCOPE screen performed in A549 cells. **a**, Hit genes identified in the screen include some single compartment and some impacting multiple compartments in the cell. Green represents hit genes called based on a subset of cell compartments (ER, mitochondria (mito), actin, DNA and Golgi/membrane) and blue represents hit genes called based on the overall profile. **b**, Hit genes called based on a single compartment are distributed across all five measured compartments. It is possible for a gene to be a hit in multiple compartments without being a whole-cell hit, see Extended Data Fig. 3a,b for more details. **c**, Distributions of optical profile correlations among all possible gene pairs versus correlations among gene pairs representing CORUM4.0 protein complexes that have at least one-third of complex subunits within hit genes. **d**, A boxen (letter-value) plot representing STRING scores divided into bins based on PERISCOPE profile correlation between gene pairs, *n* = 1,089 genes. The boxen plots display the data as a distribution where the center line represents the median, the central box represents the interquartile range from 25th to 75th percentile and the subsequent boxes represent increasingly narrower quantiles calculated for half of the remaining data. **e**, UMAP embedding of the hit gene profiles from the A549 dataset. Each dot represents a genetic perturbation and distance implies the correlation of profiles in a two-dimensional embedding. Manual annotation of cluster functions is presented for highlighted clusters based on GO datasets. Example insets show the coherent clustering of related genes. **f**,**g**, Heat maps representing Pearson correlation between gene profiles after hierarchical clustering using Ward’s method: gene complexes/processes were enriched in the A549 dataset based on the preranked GSEA analysis and show hit genes belonging to the GO:BP microtubule nucleation genes set (GO:0007020) (**f**) and hit genes belonging to the GO:BP histone modification (GO:0016570) (**g**).

**Extended Data Fig. 8:**
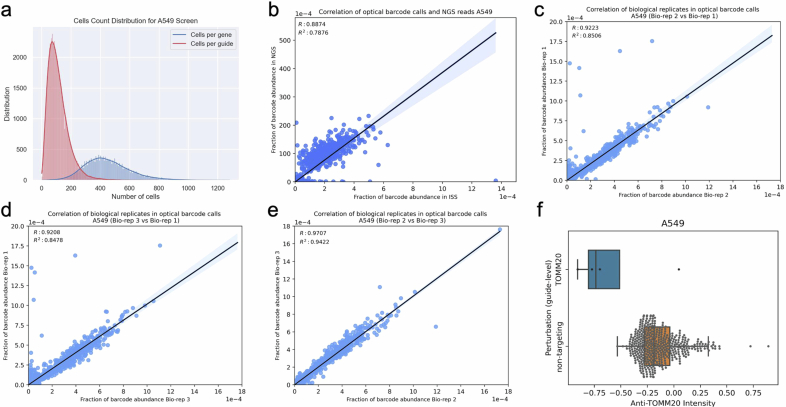
Technical summary of the A549 whole genome screen. (**a**) The distribution for the number of cells per gene and per guide present in the A549 dataset (not including nontargeting guides). (**b**) Comparison of the relative abundance of barcodes as quantified by NGS or in situ sequencing (R^2^ = 0.84), n=65,000. (**c-e**) Comparison of the relative abundance of barcodes as quantified by in situ sequencing among 3 different biological replicates representing individual viral transductions (R_1to2_^2^ = 0.85, R_1to3_^2^ = 0.85, R_2to3_^2^ = 0.94), n=80,000. The correlation coefficients in (**b-e**) are calculated using Pearson correlation, and the solid black line represents a linear regression fit of the data, with the shaded region around the regression line indicating the 95% confidence interval calculated using the standard error of the regression. (**f**) The distribution of normalized mean intensity in the mitochondrial channel from guide aggregated profiles in the A549 dataset. Every dot overlaid on the boxplots represents a sgRNA (n=4 for guides targeting the TOMM20 gene and n=450 for nontargeting guides). The boxplots display the data as a distribution where the box spans from the first to the third quartile with the median as the center line. The whiskers extend to the maximum range of the distribution within 1.5 times the interquartile range.

After successfully completing our first two whole-genome screens, we wanted to maximize the extensibility of our next whole-genome dataset by using A549 human lung cancer cells, a cell line commonly used for Cell Painting^46–48^. This decision was driven by the morphological profiling field’s active curation^19^ of Cell Painting datasets and ongoing efforts by other laboratories to develop alignment methods^49^. Using 54 identically prepared wells of six-well plates, we collected morphological profiles from 11,211,357 single cells, which yielded 20,393 gene-level profiles at an average representation of 460 cells per gene (s.d. of 707) and 117 cells per guide (s.d. of 354) (excluding nontargeting controls). Technical quality metrics such as representation, barcode calling and NGS concordance and biological replicate concordance were comparable to those of the HeLa screens (Extended Data Fig. 8). We again thresholded hits (Fig. 4a) and identified compartment hits from all subcellular compartments (Fig. 4b and Extended Data Fig. 4e,f) and, as in the HeLa screens, we found that physically interacting proteins (per CORUM and STRING) were more likely to have similar morphological profiles than random hit gene pairs (Fig. 4c,d).

Unbiased clustering of screen hits based on morphological similarity revealed logical groupings by biological function, spanning processes such as glycosylation, autophagy, proteasomal protein catabolic processes, mRNA processing, ribosomal RNA metabolic process, noncoding RNA metabolic process and mitotic cell cycle (Fig. 4e). Hierarchical clustering based on high-dimensional profiles also revealed biologically coherent clustering of perturbations targeting related genes such as those involved in microtubule nucleation (Fig. 4f) and histone modification (Fig. 4g).

**Extended Data Fig. 9:**
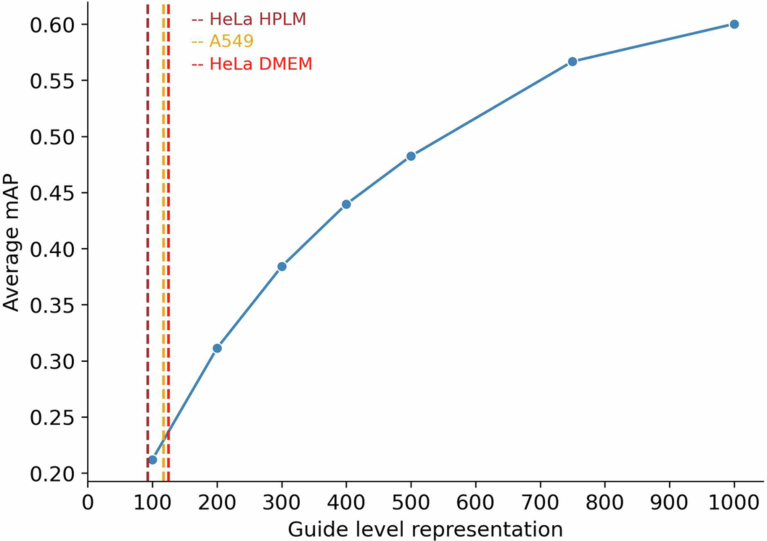
Guide representation affects profile strength and similarity in pooled CRISPR screens. Mean average precision (mAP) was calculated at different representation levels subsampled from Funk et al.^10^ by scoring each guide's ability to retrieve other guides targeting the same gene from the pool of all non-targeting guides based on cosine similarity (see Methods for calculation). mAP is a proxy for profile strength and similarity. Highlighted points represent mAP at specified mean guide-level representation from the PERISCOPE datasets for comparison.

Although we were able to extract meaningful biology from our A549 dataset, we were initially surprised that it displayed noticeably lower overall signal than our HeLa datasets, despite similar cell coverage (cells per sgRNA). Further examination revealed reduced CRISPR efficiency in our A549 Cas9 cell line compared with HeLa (~60% versus ~90%, as measured by indel sequencing), leading to reduced effective cell coverage in this screen. To further investigate the relationship between cell coverage and signal, we subsampled data from Funk et al.^10^, a highly sampled (>1,000 cells per sgRNA) optical pooled CRISPR screen and found that guide-level representation strongly affects profile strength^29^ (Extended Data Fig. 9). Despite differences in screening time point and phenotypic readout between this study and the PERISCOPE screens, this observation suggests that our screens have not reached signal saturation and that increasing cell coverage could enhance our ability to detect perturbation phenotypes.

### Genome-wide screens for subcellular phenotypes of interest

**Fig. 5:**
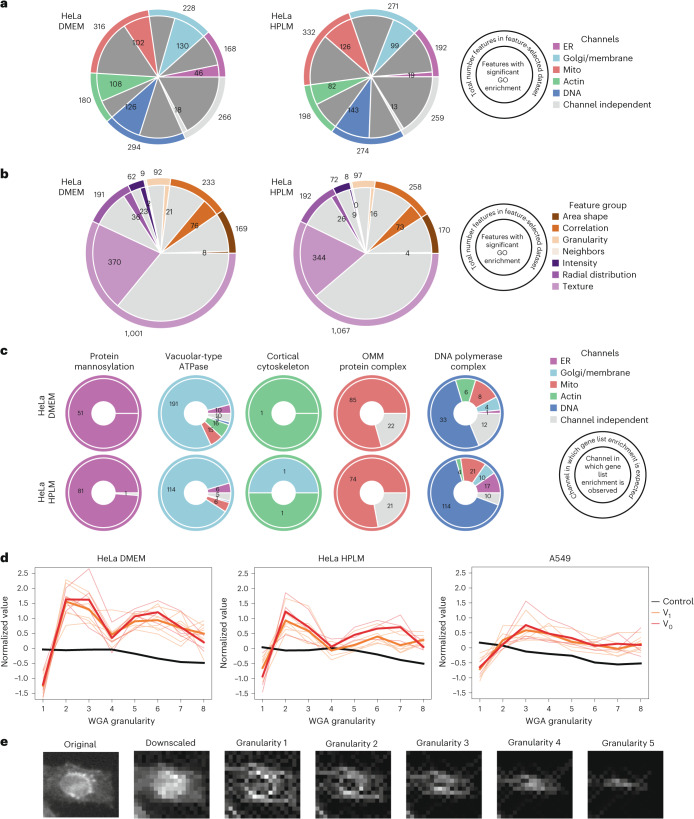
Identifying biological pathways using individual subcellular image features. **a**, GO enrichment is found in many individual features in a manner that is fairly evenly distributed across the cellular structures (that is, channels) imaged in PERISCOPE. The outer ring is the total number of features in our feature-selected dataset. The inner ring is the number of features that show GO enrichment. **b**, GO enrichment in individual features is not distributed evenly across classes of features. The outer ring is the total number of features in our feature-selected dataset. The inner ring is the number of features that show GO enrichment. **c**, Given gene groups whose protein products are expected to function specifically in a cellular structure imaged in PERISCOPE are specifically enriched in hit lists for features in those compartments. The outer ring indicates the channel in which enrichment is expected. The inner ring is the breakdown of actual channels that show enrichment for the gene group. **d**, Disruption of the vacuolar ATPase (either V_0_ or V_1_ subunit) causes a specific decrease in the screen feature WGA granularity 1, with compensatory increases across larger granularities. Each trace is a single gene. The bold lines are the mean of all genes in the group. Only hit genes are plotted. **e**, An example visualization of the signal measured at each granularity is shown for a single cell in the WGA channel.

High-dimensional profiles generated by PERISCOPE are composed of thousands of individual phenotypic features, capturing comprehensive information about stains in each channel (for example, correlation, granularity, intensity, radial distribution and texture features) for identified objects (cells, cytoplasm and nuclei), with a subset also measured on a per-image basis. Additional features describe objects (area shape features) and their relationship to nearby objects (neighbors features). Having seen that full morphological profiles capture biologically meaningful patterns of similarity, we next explored whether the datasets could be used to conduct genome-wide screens for individual morphological phenotypes of interest. To explore the single-feature screen space, we analyzed each feature in our feature-selected datasets, generating a most-perturbed gene list and assessing Gene Ontology (GO) enrichment within that list. Features with GO enrichment were distributed across imaging channels for both HeLa screens (Fig. 5a), which is unsurprising given that each channel contributed similarly to our profile-based hit lists (Figs. 2b and 3b) and all canonical channels contribute fairly evenly to profile strength in the Cell Painting assay^20^. Features with GO enrichment were not as evenly distributed across feature classes (Fig. 5b). The texture class, which had the most features, also had the highest proportion of its features (37%: 370 out of 1,001 features) enriched for a GO term. However, it is likely that many of these features are somewhat correlated since our feature selection step removes only the most highly correlated features. Notably, area shape and intensity features, which are often emphasized in other studies because of their biological interpretability, were less specifically enriched than less readily understandable categories such as texture and correlation.

To support the validity of the dataset’s single-feature screens, we looked at groups of genes whose protein products are known to function in the compartments that we labeled in PERISCOPE and determined which features had hit lists enriched for those groups. Figure 2c shows that perturbing these groups of genes produces signal across the channels, while Fig. 5c demonstrates specific enrichment in our hit lists for features in expected categories for protein mannosylation, vacuolar-type ATPase, cortical cytoskeleton and outer mitochondrial membrane (OMM) protein complex. Unsurprisingly, perturbation of DNA polymerase generated a more pleiotropic phenotype. Feature enrichment was similar between HeLa DMEM and HPLM screens (Fig. 5a–c), but the relatively reduced strength of the A549 screen resulted in negligible enrichment.

**Extended Data Fig. 10:**
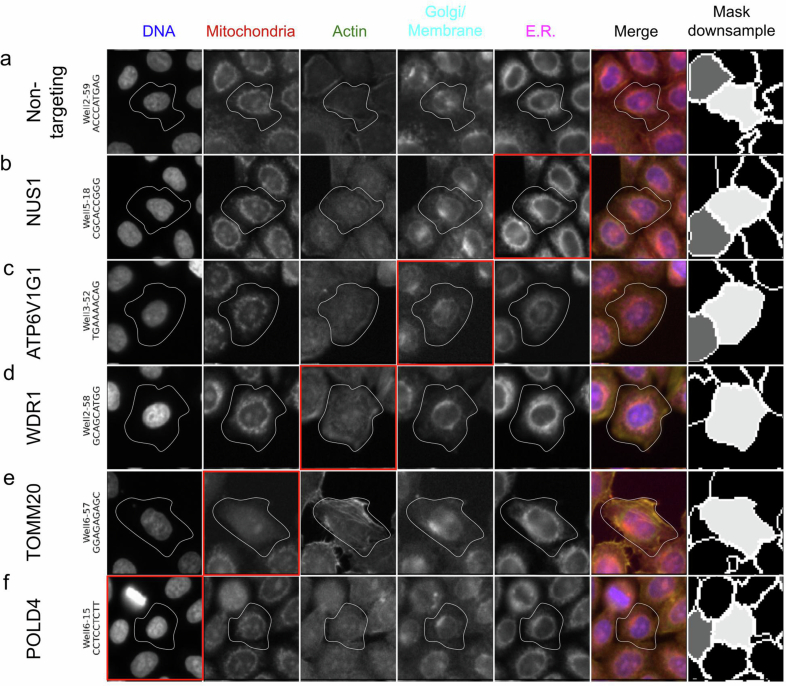
Examples of single cell images with strong morphological profiling phenotypes detected in individual channels. Representative single cell images showing each of the acquired channels, a five-color merge, and cell mask for non-targeting control (**a**) and five specific perturbations (**b-f**) from the HeLa DMEM dataset. Representative perturbations from gene sets highlighted in Fig. 2c were selected for having a large number of significantly perturbed features in a specific channel (red box) and therefore showing a strong phenotype by morphological profiling that may or may not be visible by eye. Representative cells are shown with light gray shading in Mask downsample panel and neighboring cells with the same perturbation are shown with dark gray shading.

Each screen dataset includes 3,973 features from our adapted Cell Painting assay. Despite considerable redundancy, particularly among texture and granularity metrics, dozens of morphological phenotypes of interest to the biological community can now be explored and hits pursued, regardless of the human interpretability of the features or a priori hypothesis. As an example, we focused on perturbations that altered granularity features^50^ in the WGA channel. Granularity measures the signal lost with successive erosions relative to the total signal, such that an increase in a granularity measurement of one size must correspond to a decrease at another size(s). Although conceptually described as a way to measure the signal present within differently sized intracellular structures, we downsampled images before quantifying erosions, making our granularity features not very human interpretable (visualized in Fig. 5e). The GO-enriched terms across the granularity features in the WGA channel present in our feature-selected dataset were overwhelmingly related to endocytic pathway acidification. This inspired us to look systematically across eight granularity erosions measured in the WGA channel in cell objects (that is, feature ‘Cells_Granularity_1_WGA’ and so on), where we found that disruption of the vacuolar ATPase (either V_0_ or V_1_ subunit) causes a decrease in WGA signal in the first granularity feature and concomitant increase in larger granularity features (Fig. 5d) for all datasets. This example highlights how, beyond morphological profiles, the individual features in our datasets can be used for hypothesis generation, though targeted follow-up experiments are required for biological interpretation. A primary advantage of image-based profiling over traditional microscopy is the quantitative and automated assessment of phenotypic features, overcoming the subjectivity of analyzing images by eye. Nonetheless, our atlas contains over 30 million individual cell images that can be evaluated for phenotypes of interest by a trained eye. To enhance the usefulness of these datasets, we developed an atlas cell retrieval tool (Methods), enabling the retrieval of individual images of cells containing perturbations of interest (Extended Data Fig. 10). Using this tool, we show that it is possible to find examples of readily interpretable image-based phenotypes, such as the depletion of TOMM20 signal in cells containing sgRNAs targeting TOMM20 (Extended Data Fig. 10e). However, most single-gene KO phenotypes, even those with strong morphological profiles, have phenotypes not readily identifiable by eye (Extended Data Fig. 10b–d,f), demonstrating the usefulness of computational feature extraction and profiling beyond simple visual inspection.

### TMEM251/LYSET is essential for lysosomal enzyme trafficking

**Fig. 6:**
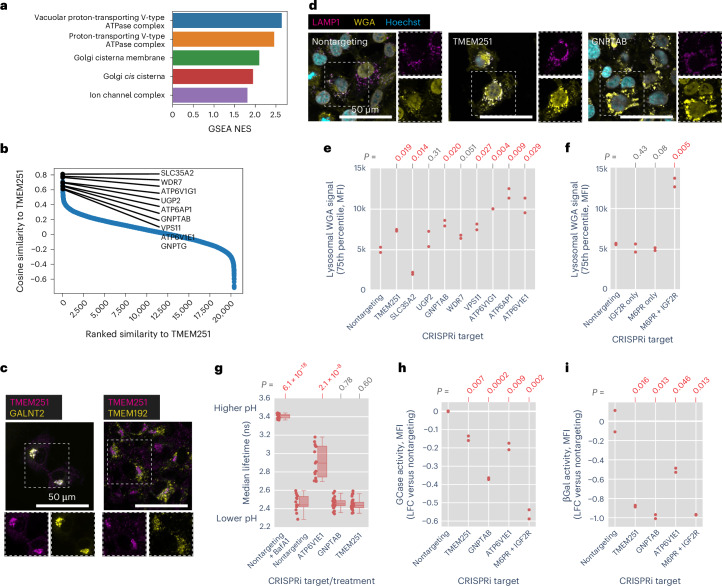
TMEM251 is essential for M6P-dependent trafficking of lysosomal enzymes. **a**, GSEA of genes preranked by cosine similarity to TMEM251 KO morphology. **b**, A waterfall plot of the distribution of cosine similarities to TMEM251 morphology. Representative genes involved in glycosylation, trafficking and lysosomal acidification are highlighted. **c**, TMEM251 localization was examined in cells expressing fluorescent reporter of either GALNT2 (Golgi) or TMEM192 (lysosome) and stained for TMEM251. **d**, WGA and LAMP1 costaining of cells with KD of genes indicated. See Supplementary Fig. 1 for other perturbations. **e**,**f**, Quantification of lysosomal WGA staining after CRISPRi KD of TMEM251, SLC35A2, UNGP2, GNPTAB, WDR7, VPS11, ATP6V1G1, ATP6AP1, ATP6V1E1 (**e**) and IGF2R and M6PR (**f**). Plotted are the upper quartiles of median per-cell lysosomal WGA intensity in two biological replicates. **g**, A box plot of LAMP1–mScarlet fluorescence lifetimes, which correlates with lysosomal pH, for the indicated perturbations. Each point represents the median lifetime of lysosomal fluorescence in an image (*n* = 30 for GNPTAB and TMEM251; *n* = 15 for the remaining conditions; boxes and mid-lines indicate Q1, Q2 and Q3, with whiskers marking the data points closest to and within 1.5× (Q3–Q1)). **h**,**i**, Log_10_ fold-changes of glucosylceramidase and beta-galactosidase activity relative to nontargeting controls for the indicated CRISPRi KDs. Each point represents the per-cell total MFI in two biological replicates. The colocalization experiment in **c** was performed once, with ~150 cells imaged over 20 fields per condition. The confocal images in **d** are representative of two biological replicates. Statistical analysis: two-tailed *t*-test versus nontargeting for **e**–**i**. βGal, beta-galactosidase; LFC, log fold-change; NES, normalized enrichment score.

Having observed that genes cluster by function using morphological profiles, we next sought to ascertain the function of uncharacterized genes based on profile similarity. We focused on the poorly characterized gene TMEM251, which clustered with genes involved in lysosomal acidification in our HeLa DMEM screen. GSEA of genes ranked by similarity to the TMEM251 KO profile in the HeLa DMEM dataset revealed enrichment for V-ATPase subunits and Golgi components, especially those related to glycosylation (Fig. 6a,b). On the basis of these term enrichments, we compared the subcellular localization of TMEM251 relative to the Golgi and lysosomes in HT1080 cells, which were selected for their relative TMEM251 growth dependency^36^. TMEM251 localized primarily to the Golgi, with negligible localization to lysosomes (Fig. 6c). TMEM251 KD with CRISPRi created strong phenotypes in the WGA channel (Fig. 6d), contributed by a striking accumulation of WGA fluorescence in LAMP1-positive lysosomes (Fig. 6d). This phenotype was seen for most of the perturbations bearing strong profile similarity to TMEM251 with the notable exception of SLC35A2, which was the most similar gene to TMEM251 at the profile level in HeLa cells, suggesting cell type-specific effects on glycoprotein accumulation in lysosomes (Fig. 6e and Supplementary Fig. 1a).

How could a Golgi-resident protein influence glycan storage in the lysosome? We postulated that the lysosomal WGA phenotype was due to impaired biogenesis of lysosomal proteins in the Golgi. Notably, GNPTAB/GPNTG showed strong phenotypic similarity to TMEM251 in PERISCOPE and human loss of function of TMEM251 results in a clinical presentation similar to that of human loss of function in GNPTAB/GNPTG^51^. We therefore hypothesized that TMEM251 may participate in the mannose-6-phosphate (M6P) pathway. In this pathway, *N*-acetylglucosamine-1-phosphate transferase (encoded by GNPTAB) attaches a phospho-GlcNac from UDP-GlcNac onto a terminal mannose that ultimately forms M6P^52^. M6P is recognized by either of two receptors, M6PR and IGF2R, and released in the lysosome in a pH-dependent manner. To further corroborate this hypothesis, we compared the phenotype of cells singly or doubly perturbed for M6PR and IGF2R. In double KD cells we observed a significant increase in lysosomal WGA accumulation, whereas single KDs were indistinguishable from wildtype, consistent with the primary screen (Fig. 6f and Supplementary Fig. 1b).

Owing to the strong morphological similarity between TMEM251 and V-ATPase subunits, we examined the effect of TMEM251 KD on lysosomal pH using a fluorescence lifetime sensor^53^. Whereas treatment with bafilomycin A1 or ATP6V1E1 KD robustly alkalinized lysosomes, neither GNPTAB nor TMEM251 KD significantly changes lysosomal pH (Fig. 6g and Supplementary Fig. 1c). We therefore reasoned that acidic lysosomal pH might be required for proper trafficking and functioning of lysosomal enzymes downstream of TMEM251’s Golgi function and that the optical profile induced by V-ATPase perturbation is dominated by this function. We tested the activity of two lysosomal enzymes that require M6PR for proper localization. Glucosyl cerebrosidase is recognized by SCARB2, which in turn interacts with M6PR to traffic to the lysosome^54^. TMEM251, GNPTAB, ATP6V1E1 and the M6PR/IGF2R double KDs all reduced glucosyl cerebrosidase activity (Fig. 6h and Supplementary Fig. 1d). Beta-galactosidase activity was even more dramatically impaired by these KDs (Fig. 6i and Supplementary Fig. 1e). During preparation of this manuscript, two independent groups reported the function of TMEM251 in the biogenesis of M6P and renamed the protein LYSET^55,56^. Our results independently support and validate a role for TMEM251 in lysosomal protein trafficking through the M6P-system.

## Discussion

Pooled optical screens are a powerful new approach for generating high-dimensional genotype–phenotype maps with single-cell resolution. Our studies demonstrate that these maps can now be generated at scale, enabling the interrogation of genome-scale perturbation effects using standard laboratory equipment (a widefield fluorescence microscope) and scalable, distributed open source analysis pipelines. Notably, the cost is remarkably low per-cell profile: ~US$0.001 per cell for the described HeLa datasets (including labor, materials and analysis, but not equipment). This combination of accessibility and cost effectiveness positions PERISCOPE-style screens as a democratizing platform technology for linking genotypes to cellular programs.

In addition to being practical, PERISCOPE generates rich, data-driven representations of gene function. A central goal of massively parallel genetic screens is to understand how genes coordinate to produce complex cell phenotypes and, in this regard, PERISCOPE is valuable both as a profiling technology—generating high-dimensional representations of a cell state—and as highly parallelized screens of subcellular biological parameters (for example, cell size and organelle size, shape and number). We showcase the ability to reconstruct relationships between genes in biological pathways and proteins in complexes using whole-cell optical profiles. Furthermore, we demonstrate the potential to gain mechanistic insights into gene function through spatially restricted subcellular phenotypes (TMEM251) and the classification of genes by function (V-ATPase assembly) using individual morphological features.

Massively parallel CRISPR modifier screens have been proven to be very useful for mapping gene-by-environment interactions at scale. By enabling facile, cost-effective genome-scale screening with high-dimensional cell profiling, we demonstrate that genetic perturbations can be readily combined with environmental perturbations to produce rich, high-resolution maps to systematically interrogate gene-by-environment interactions at genome scale. As an example, we show how such maps can uncover media-specific effects on cellular programs, but we additionally envision using this platform to execute genome-wide screens for modifiers of therapeutic compound-induced phenotypes, or to carry out genetically anchored CRISPR screens^57^ to elucidate genetic interaction networks.

### Limitations, improvements and future applications

Now that the PERISCOPE technique is established, much can be done to further optimize the workflow such that it is a routine assay. The current labor required is strongly correlated with the number of plates processed. The enzymatic, staining and imaging steps take around 4 weeks for two scientists with access to two microscopes to complete a nine-plate A549 whole-genome screen. Image analysis and profile generation require at least another 2 weeks with existing parallelization. The number of plates is affected by cell size (for example, an A549 screen required roughly twice the number of plates as an HeLa screen) and target cell coverage (a higher representation improves the signal to noise ratio to enable detection of more subtle perturbations, and lower Cas9 efficiency requires higher representation). Automation of both wet laboratory and computational workflows has the potential for a profound impact on throughput. If experimental modifications that reduce throughput are required, such as higher magnification for imaging phenotypes, we suggest a compensatory modification such as focusing only on expressed genes or using vector systems that reduce the number of guides required.

In addition to improving cell coverage, the signal in PERISCOPE screens can be further improved by refining the background distribution through careful curation of negative control perturbations. Here, we use nontargeting sgRNAs to identify gene-targeting sgRNAs that produce significant morphological signal (a standard practice in CRISPR screening^2^), and then take the further step of using all sgRNAs targeting nonexpressed genes (Zero-TPM in the DepMap database) to apply stringent FDR correction to our hit list. While this conservative approach attempts to reduce the signal from a wide range of nonspecific morphological effects associated with CRISPR cutting (as opposed to gene-specific KO effects), it relies on the accuracy of underlying expression data. As we observe, genes with very low expression can still produce morphological phenotypes when perturbed and, additionally, fitness effects can be induced by gene-independent activity of sgRNAs targeting amplified genes^58^, dampening the screen signal. The use of a curated set of intergenic cutting sgRNAs could mitigate this effect while still reducing nonspecific signal from CRISPR activity. On a related note, though we have applied a strict 1% FDR threshold to our data, we encourage users of these open source data to apply their own judgment when selecting a FDR to balance the ratio of false positives/negatives based on their specific applications (for example, discovery versus validation).

Beyond the current scope, there are several improvements that could be built upon the foundation of the work presented here. In its current form, the PERISCOPE platform could be deployed to explore the effects of other CRISPR-based perturbations such as CRISPR-a^59,60^, CRISPR-i^61,62^ or base editing^63–65^, where sgRNAs can be expressed as an RNA Pol II transcript (as in CROP-seq). In this study, we profile two cancer cell lines, HeLa and A549, but our pipelines are amenable to screening a wide variety of two-dimensional cell models, including cell lines and primary cells, though assay scale and data quality are cell density dependent. Our screens demonstrate that significant signal is present in every measured cell compartment, and highly multiplexed imaging technologies such as CODEX^66^ and CyCIF^67^ could improve the sensitivity and robustness of PERISCOPE by capturing a wider range of perturbation effects or enabling the inclusion of ground truth epitopes to anchor biological interpretation. Extracting biological signals from fluorescence multicolor images is a compelling machine learning problem, which will probably be improved using various forms of deep learning, such as self-supervised learning, to extract features^68^. Though such features lack inherent interpretability, which is important for some applications, they have proven to be more powerful than engineered features for capturing similarities in some cases^69,70^.

Though we are able to extract meaningful biology from our datasets, it is clear that our current cell coverage somewhat limits the biology that can be extracted from our proof-of-principle datasets and that increased cell sampling should be considered for future PERISCOPE screens, including improvements to the computational workflow such that improved barcode calls^71^ and cell assignments result in fewer cells being filtered out. That being said, beyond the biological validation we report here, new methods for quantifying signal in large-scale screens validate that we have clear signal in our HeLa datasets^72^ and that they can outperform many other datasets as sources of prior information for predicting the outcome of Perturb-seq experiments^73^, supporting the utility of this resource.

In sum, this study lays the groundwork for building high-dimensional morphology-based perturbation maps at scale and presents the first genome-scale atlas of human cell morphology. Containing more than 30 million perturbation-assigned cell images, this atlas is a useful resource for biological interrogation as well as for the development and testing of new computational image analysis methods. All data and analysis tools are open source and freely available (Code availability and Data availability).

## Methods

### Library design

The whole-genome library was designed to target 20,393 genes with ~4 sgRNAs per gene for a total of 80,408 sgRNAs. Guides were selected from a larger set (20 sgRNAs per gene) that was computationally designed by the Broad Institute’s Genetic Perturbation Platform to optimize predicted editing efficiency while ensuring that individual guides were distinguishable by at least 2 bases in their first 12 nucleotides (to facilitate error detection during ISS). Among the 80,408 sgRNAs, 47,792 sgRNAs are present in the Brunello CRISPR library (Addgene, 73179) and 20,520 sgRNAs are in the TKO V3 CRISPR library (Addgene, 90294). Additionally, 601 nontargeting sgRNAs were included as negative controls. All sgRNA sequences were selected/designed to maintain a balanced nucleotide distribution at each base position, which facilitates optical barcode calling. The CRISPR library was designed for complete library deconvolution with 11 bases and for Levenshtein error correction with 12 bases.

### Library cloning

To prepare pooled plasmid libraries, targeting and nontargeting guide subpools were first individually amplified by dial-out PCR using orthogonal primer pairs.^74^. PCR products were purified using the QIAquick PCR purification kit (Qiagen, 28104). The amplified libraries were cloned into the CROP-seq vector (Addgene, 86708) via Golden Gate assembly using BsmBI restriction sites as previously described^13^. To prevent self ligation events in Golden Gate reactions, the CROP-seq vector was predigested and purified via gel extraction using the QIAquick gel extraction kit (Qiagen, 28706) to remove the filler sequence. The resulting plasmid libraries were purified and concentrated via solid-phase reversible immobilization bead cleanup before being transformed into electrocompetent cells (Lucigen Endura, VWR International, 71003-038) for plasmid library amplification. Following transformation, bacterial cells were grown in liquid cultures for 18 h at 30 °C before extracting the plasmid DNA. The plasmid library was validated via NGS as described in NGS.

### Tissue culture

A549 cells were cultured in high-glucose DMEM (VWR International, 45000-304) supplemented with 2 mM l-glutamine (Life Technologies, 25030081), 100 U ml^−1^ penicillin–streptomycin (Life Technologies, 15140163) and 10% heat-inactivated fetal bovine serum (FBS) (Sigma-Aldrich, F4135-500ML). HEK293FT cells were cultured in DMEM–GlutaMax, pyruvate (Thermo Fisher Scientific, 10569010) supplemented with 10% heat-inactivated FBS and 100 U ml^−1^ penicillin–streptomycin and 2 mM l-glutamine. HEK293FT cells were also cultured without antibiotics 24 h before lentiviral packaging. HeLa cells in the conventional media screen were cultured in DMEM (VWR International, 45000-304) supplemented with 10% dialyzed FBS (Thermo Fisher Scientific, 26400044). HeLa cells in the physiological media screen were cultured in HPLM (Thermo Fisher Scientific, A4899101) supplemented with 10% dialyzed FBS.

### Lentivirus production

Before lentivirus production, the plasmid pools for targeting and nontargeting sgRNAs were combined resulting in a 10% (mass/mass ratio, m/m) of nontargeting sgRNAs and a 90% (m/m) of targeting sgRNAs. At 24 h before transfection, HEK293FT cells were seeded on 10 cm^2^ dishes at a density of 100,000 cells cm^−2^ using antibiotic-free medium. The lentivirus was generated using the Lipofectamine 3000 (Thermo Fisher Scientific, L3000015) transfection kit and packaging plasmids pMD2.G (Addgene, 12259) and psPAX2 (Addgene, 12260). HEK293FT cells were transfected with a plasmid ratio of 2:3:4 (by mass) of pMD2G, psPAX2 and plasmid library, respectively. Media were exchanged 4 h after transfection. The lentivirus was collected 48 h after media exchange and filtered through a 0.45 µm cellulose acetate filter (Corning, 431220). The viral supernatant was incubated in dry ice until frozen and stored at −80 °C.

### Lentivirus titering

A viral titer was individually determined for A549 and HeLa cells. A549 cells were seeded at a density of 100,000 cells cm^−2^ while HeLa cells were seeded at a density of 150,000 cells cm^−2^ in a 6-well format. The seeded cells were transduced with the viral library by supplementing their media with 8 μg ml^−1^ of polybrene (Sigma-Aldrich, TR-1003) and adding a variety of viral volumes ranging from 0 μl to 50 μl before centrifugation at 1,000*g* for 2 h at 33 °C. After centrifugation, the cells were incubated at 37 °C for 4 h followed by a media exchange. At 24 h post-infection, cells were divided into media containing either 0 μg ml^−1^ or 2 μg ml^−1^ of puromycin (Life Technologies, A1113803). Cells in both media conditions were incubated at 37 °C for 72 h. Following incubation, cells were counted and multiplicity of infection (MOI) was estimated by the ratio of surviving cells in the 2 μg ml^−1^ puromycin conditions over puromycin free conditions. Infectious units per microliter (ifu μl^−1^) were then calculated by multiplying the MOI by the original cell seeding density and dividing by the viral volume added. The values of ifu μl^−1^ for each viral volume were averaged and used to estimate viral volume required to achieve an MOI between 0.1 and 0.3.

### Lentivirus transduction

For screens, cells were transduced with the genome-wide viral library in a 6-well format by adding 8 μg ml^−1^ of polybrene and the volume of viral supernatant calculated for an MOI of 0.2 as well as a noninfection control with 0 μl of viral supernatant. Cells were centrifuged at 1,000*g* for 2 h at 33 °C. At 4 h post-infection, media were exchanged. At 24 h post-infection, the infected cells were passaged into T-225 flasks (VWR International, 47743-882) containing media supplemented with 2 μg ml^−1^ puromycin. A fixed number of cells (~300,000) for the infection and uninfected conditions were set aside and seeded in a 6-well plate format under media containing either 0 μg ml^−1^ or 2 μg ml^−1^ of puromycin. All cells were incubated at 37 °C for 72 h. Following the 72 h of selection, the cells seeded in the 6-well plate were counted and the MOI was calculated as described above.

### A549 screen

A549-TetR-Cas9 cells were transduced with the genome-wide viral library in three biological replicates by seeding cells at a density of 150,000 cells cm^−2^ in a 6-well format and performing lentiviral transduction as described above. A total of 240,000,000 cells were transduced at an MOI of 0.2 for a cell library representation of 300 cells per sgRNA post transduction. After antibiotic selection, the cells were cultured in conventional DMEM media for 2 days. Before induction of Cas9 expression, a sample of 25,000,000 cells per biological replicate were lysed and prepared for NGS as described below. These samples were used to confirm the target representation. Cas9 expression was induced with 2 μg ml^−1^ doxycycline spiked in conventional DMEM medium. Throughout Cas9 expression, cells were cultured in T-225 flasks and passaged once the flasks reached 70% confluency. Between passages, a minimum of 24,000,000 cells were re-seeded per biological replicate thus maintaining a representation of 300 cells per sgRNA. The cells were supplemented with 2 μg ml^−1^ of doxycycline every 2 days by exchanging the culturing media. On day 5 of Cas9 expression, the cells were seeded into nine 6-well glass-bottom plates (Cellvis, P06-1.5H-N) at a density of 19,800 cells cm^−2^. A total of 13,000,000 cells across the three biological replicates were seeded in optical plates with the expectation that cell populations will double at least once before fixation. The remainder of the cells were kept in T-225 flasks and cultured until day 7 of Cas9 expression where a sample of 13,500,000 cells from each biological replicate were lysed and prepared for NGS analysis. At 48 h after being seeded in optical plates, the cells were fixed with 4% paraformaldehyde in 1× PBS for 30 min, followed by ISS as described below. After rolling circle amplification (RCA) in ISS, the cells were stained with cell compartment-specific probes as described in ‘Phenotypic labeling’ and phenotypic images were acquired. The disulfide-linked probes were destained by cleaving the disulfide bridge between the probe and its fluorophore with 50 mM TCEP (Thermo Fisher Scientific, 363830100) in 2× saline–sodium citrate (SSC) for 45 min at room temperature.

After destaining phenotypic probes, the cells are washed three times with 1× PBS-T (1× PBS + 0.05% Tween-20) before performing 12 cycles of ISS.

### HeLa screens

HeLa-TetR-Cas9 were transduced with the genome-wide viral library in three biological replicates by seeding cells at a density of 210,000 cells cm^−2^ in a 6-well format and performing lentiviral transduction as described above. A total of 240,000,000 cells were transduced at an MOI of 0.2 for a cell library representation of 300 cells per sgRNA post transduction. After antibiotic selection, the transduced cells were cultured in conventional DMEM medium until a representation of 600 cells per sgRNA was achieved. To confirm the target representation, a sample of 20,000,000 cells from each biological replicate were lysed and prepared for NGS as described below. The cell library was then divided into two culturing conditions, conventional DMEM and physiological HPLM media (media formulations are described above). Simultaneous to the addition of these two media conditions, Cas9 expression was induced with 2 μg ml^−1^ doxycycline (Sigma-Aldrich, D5207) for 7 days. Throughout Cas9 expression, cells for each condition were cultured in T-225 flasks and passaged once the flasks reached 70% confluency. Between passages, a minimum of 24,000,000 cells were re-seeded per biological replicate thus maintaining a representation of 300 cells per sgRNA for each media condition. The cells were supplemented with 2 μg ml^−1^ of doxycycline every 2 days by exchanging the culturing media. On day 5 of Cas9 expression, the cell libraries under both media conditions were seeded into five 6-well glass-bottom plates (Cellvis, P06-1.5H-N) at a density of 42,000 cells cm^−2^. A total of 14,000,000 cells across the three biological replicates were seeded in optical plates for each media condition with the expectation that cell populations will double at least once before fixation. The remainder of the cells were kept in T-225 flasks and cultured until day 7 of Cas9 expression, where a sample of 20,000,000 cells from each biological replicate was lysed and prepared for NGS analysis. At 48 h after being seeded in optical plates, the cells were fixed with 4% paraformaldehyde in 1× PBS for 30 min, followed by ISS as described below. After RCA amplification in ISS, the cells were stained with cell compartment-specific probes as described in ‘Phenotypic labeling’ and phenotypic images were acquired. The disulfide-linked phenotypic probes were destained by cleaving the disulfide bridge between the probe and its fluorophore with 50 mM TCEP (Thermo Fisher Scientific, 363830100) in 2× SSC for 45 min at room temperature. After probe destaining, the cells are washed three times with 1× PBS-T (1× PBS + 0.05% Tween-20) before performing 12 cycles of ISS.

### Synthesis of destainable phenotyping probes

Due to the spectral overlap between the fluorescent dNTPs required for ISS and the available fluorophores for phenotypic markers, the probes used to label the mitochondria and the ER were synthesized in-house to include a disulfide bridge between the probe and its fluorophore that will allow for cleavage of the fluorophore after imaging. For mitochondria labeling, the secondary anti-TOMM20 antibody, F(ab’)2-goat-anti-rabbit IgG (H + L) (Thermo Fisher, 31239) was conjugated to Alexa Fluor 594-azide (Thermo Fisher, A10270). For ER labeling, the protein ConA (Sigma-Aldrich, C2010) was conjugated to cyanine 5-azide (Lumiprobe, B3030). In the synthesis of these probes, we leveraged the thermal stability and high specificity of the click chemistry reaction between dibenzocyclooctyne (DBCO) and azide groups. Hence, the anti-TOMM20 antibody and the ConA protein were functionalized for click chemistry with the addition of an NHS-SS-DBCO molecule (Sigma-Aldrich, 761532) that subsequently reacted with the azide groups linked to their respective fluorophores. Before functionalizing the probes, the anti-TOMM20 antibody and ConA protein were diluted to 1.1 mg ml^−1^ and 2 mg ml^−1^ in freshly prepared 0.1 M sodium phosphate solutions at pH 8.5 and 6.8, respectively. The DBCO was freshly dissolved to 10 mg ml^−1^ in anhydrous dimethylsulfoxide (Sigma-Aldrich, 227056). The diluted proteins and DBCO were combined at the following molar rations (8 anti-TOMM20:1 DBCO and 3 ConA:1 DBCO) and then incubated for 2 h at 4 °C while shaking. Following incubation, the reaction was quenched with 2 M Tris–HCl (pH 7.4) at a 10% reaction volume. The resulting product was purified using Zeba columns (Thermo Fisher, 89883). Product retention after column purification was ~90%. The azide-linked fluorophores were diluted to 10 mg ml^−1^ in anhydrous dimethylsulfoxide and reacted with their respective functionalized probes at a 3:1 molar ratio. This reaction proceeded for 20 h at 4 °C while shaking; reaction vials were protected from light during this incubation. The final product was purified by running each reaction through three Zeba columns to do a final buffer exchange into 1× PBS. After synthesis the destainable probes were stored at −20 °C.

### ISS

The ISS of sgRNAs required three enzymatic steps, a targeted reverse transcription (RT) of the sgRNA, the formation of a circular DNA template (gap-fill and ligation) and the amplification of that template through RCA. Before the enzymatic reactions, cells were fixed with 4% paraformaldehyde (Electron Microscopy Sciences, 15714) in 1× PBS for 30 min at room temperature and then permeabilized with 70% ethanol (VWR International, 76212-358) for 30 min at room temperature. To prevent sample dehydration after permeabilization, the ethanol was removed over six serial dilutions with PBS-T (1× PBS + 0.05% Tween-20). After permeabilization, the RT solution was prepared and applied to the cells according to the following formulation of 1× RevertAid RT buffer (Thermo Fisher, EP0452), 250 μM dNTPs (NEB, N0447L), 0.2 mg ml^−1^ BSA (NEB, B9000S), 1 μM RT primer (G + AC + TA + GC + CT + TA + TT + TTAACTTGCTAT), 0.8 U μl^−1^ Ribolock RNase inhibitor (Thermo Fisher, EO0382) and 4.8 U μl^−1^ RevertAid H minus reverse transcriptase (Thermo Fisher, EP0452). Cells in the RT solution were incubated at 37 °C overnight.

Following RT, the cells were washed five times with PBS-T and post-fixed with 3% paraformaldehyde and 0.1% glutaraldehyde (Electron Microscopy Sciences, 16120) in 1× PBS for 30 min at room temperature. After post-fixation, the cells were washed three times with PBS-T. The gap-fill and ligation solution was prepared and added to the cell according to the following formulation of 1× Ampligase buffer (Lucigen, A3210K), 50 nM dNTPs (NEB, N0447L), 0.2 mg ml^−1^ BSA (NEB B9000S), 10 nM padlock probe (/5Phos/ GTTTTAGAGCTAGAAATAGCA AGCTCCTGTTCGACACCTACCCACCTCATCCCACTCTTCAAAAGGACGAAACACCG), 0.4 U μl^−1^ RNase H (Qiagen, Y9220L), 0.002 U μl^−1^ TaqIT polymerase (Qiagen, P7620L) and 0.5 U μl^−1^ Ampligase (Lucigen, A1905B).

After gap-fill and ligation, the cells were washed three times with PBS-T. The RCA solution was then prepared according to the following formulation of 1× Phi29 buffer (Thermo Fisher, EP0094), 250 μM dNTPs (NEB, N0447L), 0.2 mg ml^−1^ BSA (NEB B9000S), 5% glycerol and 1 U μl^−1^ Phi29 DNA polymerase (Thermo Fisher, EP0094). The cells in the RCA solution were incubated at 30 °C overnight. Following incubation, the cells were washed three times with PBS-T.

### Phenotypic labeling

After RCA, the cells were prepared for phenotypic labeling by incubating them with a blocking buffer containing 1% BSA (Seracare Life Sciences, 1900-0016) in 1× PBS for 10 min at room temperature. After blocking, a primary staining solution containing rabbit anti-TOMM20 antibody (Abcam, ab78547), Alexa Fluor 488 Phalloidin (Thermo Fisher, A12379), ConA-SS-A647 and WGA-A750 (WGA protein by Vector Labs, L-1020-20, custom conjugation to A750 fluorophore by Arvys Proteins) was prepared in 1× PBS and applied to the cells for 45 min at room temperature. Following incubation with the primary staining solution, the cells were washed three times with 1× PBS-T and a secondary staining solution containing F(ab′)2-goat-anti-rabbit IgG (H + L)-SS-A594 was prepared in blocking buffer and applied to the cells for 30 min at room temperature. The phenotypic probes for the primary and secondary staining solutions were diluted according to the dilution factors listed in Supplementary Table 4. Dilution factors for each probe were determined before screening by doing a serial titration of individual stains.

After incubation with the secondary staining solution, the cells were washed with 1× PBS-T three times allowing the plate to sit at room temperature for 5 min between washes. Finally, the cells were placed in a freshly prepared DAPI staining solution containing 200 ng ml^−1^ DAPI (Sigma-Aldrich, D9542-10MG) diluted in 2× SSC. The cells were incubated in the DAPI staining solution for 10 min at room temperature before imaging.

### Sequencing by synthesis

After destaining the phenotypic probes, the cells were incubated with a sequencing primer (CACCTCATCCCACTCTTCAAAAGGACGAAACA CCG) at 1 μM concentration in 2× SSC with 10% formamide for 30 min at room temperature. Following this primer hybridization, the cells were washed three times with PR2 buffer (Nano kit PR2) and then incubated with incorporation mix (Nano kit reagent 1) for 5 min at 60 °C. The incorporation mix was then removed over six serial dilutions with PR2 buffer. To decrease background fluorescence, the cells were washed with fresh PR2 buffer and incubated at 60 °C for 5 min. The washing process was repeated five times before adding 200 ng ml^−1^ DAPI (Sigma-Aldrich, D9542-10MG) in 2× SSC and imaging.

### Fluorescence microscopy

Phenotypic and ISS images were acquired using a Nikon Ti-2 Eclipse inverted epifluorescence microscope with automated *XYZ* stage control, an Iris 9 scientific complementary metal–oxide–semiconductor (sCMOS) camera (Teledyne Photometrics) and hardware autofocus. All hardware was controlled using NIS-Elements AR, and a CELESTA light engine (Lumencor) was used for fluorescence illumination. Phenotypic images were acquired using a 20× 0.75 numerical aperture (NA) chrome-free infinity corrected (CFI) Plan Apo Lambda objective (Nikon, MRD00205) and the following Semrock filters for each phenotypic probe: actin (phalloidin) emission ET530/30 nm, dichroic 495 nm; mitochondria (TOMM20) emission 615/24 nm, dichroic 565 nm; ER (ConA) emission 680/42 nm, dichroic 660 nm; Golgi and plasma membrane (WGA) emission 820/110 nm, dichroic 765 nm; nucleus (DAPI) dual-band emission 408/473, dichroic 408/473 nm. ISS cycles were imaged using a 10× 0.45 NA CFl Plan Apo Lambda objective (Nikon) with the following Semrock filters for each base: Miseq G excitation 543/4 nm, emission 575/30 nm, dichroic 555 nm; Miseq T emission 615/24 nm, dichroic 565 nm; Miseq A emission 680/42 nm, dichroic 660 nm; Miseq C emission 732/68 nm, dichroic 660. Laser power for all acquisitions was kept at 30%. The exposure times for ISS cycles were selected by balancing the average pixel intensities of ISS spots in each fluorescent channel.

### NGS

NGS was used for validation of plasmid libraries, cell libraries and Cas9 activity in screening cell lines. For Cas9 activity assays and cell library validation, cell samples were lysed by resuspending cell pellets in lysis buffer (10 mM Tris pH 7.5, 1 mM CaCl_2_, 3 mM MgCl_2_, 1 mM EDTA, 1% Triton-X100 and 0.2 mg ml^−1^ Proteinase K) and heating for 10 min at 65 °C followed by 15 min at 95 °C. The target sequences in cell lysates were directly amplified without cell lysis purification according to the following PCR reactions: PCR 1: 1× Kappa HiFi, 0.15 µM CROP-seq-puro P5 (CTGGAGTTCAGACGTGTGCTCTTCCGATCaagcaccgactcggtgccac), 0.15 µM CROP-seq-puro P7 (ACACGACGCTCTTCCGATCTtcttgtggaaaggacgaaac), 2 ng µl^−1^ gDNA from cell lysate, 28 PCR cycles. PCR 2: 1× Kappa HiFi, 0.25 µM P5 Truseq Indexing Primer FWD, 0.25 µM P7 Truseq Indexing Primer RVD, 4 ng µl^−1^ PCR 1 product, 18 PCR cycles. Temperature conditions for PCR reactions followed initial denaturation at 95 °C for 5 min, then denaturation at 95 °C for 20 s, annealing at 55 °C for 30 s and extension at 72 °C for 30 s. PCR 2 products were purified via gel extraction using the Qiaquick gel extraction kit (Qiagen, 28706×4) and prepared for sequencing as described in Illumina’s library denaturation and dilution manual. The PhiX Control library was spiked in the sequencing sample at 10% (v/v) (Illumina, FC-110-3001).

### Cell lines

The A549-TetR-Cas9 cell line^75^ was created by simultaneously transfecting A549 cells with *piggyBac* transposase (HP137) and a *piggyBac* cargo plasmid containing TetR-inducible Cas9 (Addgene, 134247), and selecting for 7 days with 500 µg ml^−1^ G418. Single cells were sorted into 96-well plates (Sony, SH800) and expanded into colonies. An optimal clone was selected on the basis of Cas9 activity, aiming for high and low activity in the presence and absence of doxycycline, respectively. Cas9 activity was evaluated using the fluorescence based reporter pXPR011 (Addgene, 59702), which expressed GFP and cognate sgRNA to assess GFP KD upon successful CRISPR activity. Fluorescence readouts of Cas9 activity were detected via fluorescence-activated cell sorting and indel sequencing. The A549 parental cells were obtained from the American Type Culture Collection (CCL-185). The HEK293FT cells used for viral packaging were obtained from Thermo Fisher Scientific (R70007). The HeLa-TetR-Cas9 cell line was a gift from Iain Cheeseman; this cell line is a single-cell clone selected for high Cas9 activity by transducing with the eGFP reporter mentioned above (pXPR011) and using fluorescence-activated cell sorting to read out efficiency of protein KD.

### Image processing

We used CellProfiler bioimage analysis software (version 4.1.3)^27^ to process the images using classical algorithms and Fiji (with openjdk-8)^76^ for image stitching^77^ and cropping. For the ISS images, we corrected for variations in background intensity, aligned channels within cycles and performed channel compensation. For the phenotypic images, we corrected for variations in background intensity. We then stitched the ISS and Cell Painting images independently into a full-well view and cropped them into corresponding pseudo-sites to account for the fact that they were imaged at different magnifications. Corrected, pseudo-site images from both ISS and phenotypic images entered our final analysis pipeline where they were aligned to each other, confluent regions (if present) were detected and masked out, nuclei and cells were segmented using phenotypic images, ISS foci were identified and a barcode was called for each focus. Then, across the various channels captured, we measured various features of cells across several categories including fluorescence intensity, texture, granularity, density and location (see http://cellprofiler-manual.s3.amazonaws.com/CellProfiler-4.1.3/index.html for more details). We obtained 3,973 feature measurements from each of about 26.8 million (A549) and 46.4 million (HeLa) cells. We parallelized our image processing workflow using Distributed-CellProfiler^78^ and Distributed-FIJI^79^, triggered by Lambda Functions in Amazon Web Services. The actual CellProfiler pipelines used are available in the Cell Painting Gallery^80^ (Code availability and Data availability) while continuously improved pipelines and Lambda Function scripts are available at https://github.com/broadinstitute/pooled-cell-painting-image-processing. Object segmentation parameters are likely to need tuning by an image analysis expert between datasets but feature extraction is invariant.

### Image-based profiling

We processed outputs of CellProfiler into image-based profiles using scripts available at https://github.com/broadinstitute/pooled-cell-painting-profiling-recipe. This is highly configurable beyond the configurations used for this report. The first step generates summaries of a variety of quality control metrics about the image acquisition, modified Cell Painting and ISS. The second step uses Pycytominer workflows to process the single-cell features extracted using Cell Profiler. We median aggregated the single-cell profiles by guide for each plate independently. Next, we defined the center and scale parameters as the mean and standard deviation of feature values by the standardized method in Pycytominer, and then normalized the averaged profiles by subtracting the center value and scaling to the standard deviation for each plate independently. We further processed the per-plate guide-level profiles to create the per-screen profiles we use in our analyses. We performed feature selection independently for each screen to eliminate noisy features and retain the most informative features by filtering out redundant features (all features that have Pearson correlation greater than 0.9 to a given feature), features with low variance, and features with missing values across all the plates as is standard in image based profiling workflows^37^. Then we median aggregated each experiment’s feature-selected per-plate profiles to obtain a unique profile per guide for each experiment. For perturbation-level (gene-level) profiles, each experiment’s guide-level profiles were median aggregated.

Each dataset is independently welded to the recipe, effectively versioning the recipe, using a template, available at https://github.com/broadinstitute/pooled-cell-painting-profiling-template. Our A549 screen data with versioned recipe are available at https://github.com/broadinstitute/CP186-A549-WG. Our HeLa screens data with versioned recipe are available at https://github.com/broadinstitute/CP257-HeLa-WG. Code used for further profile processing is in this paper repository at https://github.com/broadinstitute/2022_PERISCOPE.

### Hit calling, statistical analysis and distribution of hits

To determine the genes with significant signal above the noise (hit calling) we developed an algorithm to compare the distribution of values per feature for all the guides targeting the same gene with a set of nontargeting control guides using the Mann–Whitney *U*-test. The number of features significantly different from the nontargeting controls based on the statistical test (*P* value of 0.001) were added up to calculate profile score for each perturbation. Then, to ensure that the perturbations called significant are truly not null, we defined a control group called zero-transcript per million (TPM) genes. Zero-TPM genes are the genes without significant expression in a given cell line and were determined based on the RNA expression levels reported by the Broad Institute Dependency Map portal^36^. To obtain a FDR of 1%, perturbations with profile scores above 99% of zero-TPM genes were determined to have significant signal above the noise. The terms ‘whole-cell hits’ and ‘compartment hits’ were used to distinguish between perturbations with significant signal in overall profile features or perturbations with targeted signal in features from a specific cell compartment (based on one of the five fluorescent markers). For whole-cell hits, all of the collected features were used in the hit-calling process explained above, but for the compartment hits, a subset of features from one cell compartment were used (including texture, intensity, correlation, radial distribution and granularity measures from that compartment). The hit-calling pipeline described above was also utilized at FDR levels 2%, 3%, 4% and 5% to highlight the number of identified hits at different stringency levels (Extended Data Fig. 3a). It is important to note that a single perturbation can be a compartment hit, targeting simultaneously two, or rarely even three, compartments, but still not be a whole-cell hit (Extended Data Fig. 4a,c,e).

### mAP calculations

Mean average precision (mAP) was used to evaluate the similarity between phenotypic profiles between guides targeting the same gene. mAP is a commonly used performance metric in machine learning, specifically for information retrieval tasks and it has been shown to be a valuable tool in validation of large-scale, high-throughout biological profiling data.^29^ From a group of *N* control profiles and a group of *M* query profiles (*M* = 4 for each guide targeting the same gene), for each query profile we calculate noninterpolated average precision (AP) by following these steps:

1. Select a single profile *i* from *M* query profiles.
2. Calculate similarity of the profile *i* to all other (*M* − 1) + *N* profiles; we have used cosine similarity as the metric.
3. Sort (*M* − 1) + *N* profiles by decreasing similarity to the profile *i*.
4. At each rank *k* going down the list, if *k* is a correct match, calculate the precision at rank *k* for this rank.
5. AP can be be calculated via relative change in recall using the following formula:
  $${\mathrm{AP}}_{i}=\mathop{\sum }\limits_{k=1}^{(M-1)+N}({R}_{k-1}-{R}_{k}){P}_{k},$$
  in which $\,{P}_{k}$ is precision and *TP*_*k*_ is true positive at rank *k*,
  $${P}_{k}=\frac{{{TP}}_{k}}{k}$$
  and ${R}_{k}$ is recall at rank *k*,
  $${R}_{k}=\frac{{{TP}}_{k}}{M-1}.$$
  Finally the mAP for the whole query group can be calculated by
  $${\mathrm{mAP}}=\frac{1}{M}\mathop{\sum }\limits_{i=1}^{M}{\mathrm{AP}}_{i}.$$
  More details and the code used to calculate mAP is available on the GitHub repository.

### Distribution of significant features based on gene sets targeting each compartment

Pie charts showing the normalized fraction of number of features significantly different from the control, categorized based on target compartments (Fig. 2c). The values are the average from multiple genes part of the highlighted gene groups.

### Comparison between pairwise correlation of perturbations to other databases

To assess the ability of phenotypic profiles to recall known biological relationships, we calculated the correlation between profiles as a measure of similarity and used it to perform two global assessments. Considering the large number of features in each profile (1,520 in A549, 1,597 in HeLa DMEM and 1,709 in HeLa HPLM datasets) and to improve the signal to noise ratio, principal component analysis (PCA) was performed on the datasets to capture at least 90% of the variation, producing 334 (A549), 325 (HeLa DMEM) and 231 (HeLa HPLM) new features. The resulting profiles were then used to calculate the Pearson correlation coefficient between all hit perturbation profiles (gene level). First, annotated protein clusters were obtained from the 28.11.2022 CORUM4.0 database^31^. Clusters with at least 33% of the hit genes were identified using the gene symbols from both datasets (645 clusters in A549, 953 clusters in HeLa HPLM and 1,350 clusters in HeLa DMEM). Then, all the correlations between each pair of genes in a cluster were calculated. The distribution of all the correlations between profiles within clusters versus the distribution of all the correlation between profiles from all hit genes were plotted in Fig. 2d. Second, we performed a similar analysis based on the protein link scores as predicted by the STRING database (v11.5, ‘9606.protein.links.v11.5.txt.gz’)^32^. To start, protein IDs from STRING were mapped to gene symbols using preferred_name extracted from the ‘9606.protein.info.v11.5.txt.gz’ file. All the possible pairwise correlations between the hit gene profile with a reported link score in the STRING database were calculated. Next, the correlations were binned into eight equally spaced bins and the distribution of the STRING link scores for each bin were plotted using seaborn.boxenplot^81^ in Python.

### Comparison to cancer dependency map data

From DepMap data, we divided genes expressed in HeLa cells into essential and nonessential categories based on DEMETER2 gene dependency scores^58^ using a threshold score of −0.5 for gene essentiality and plotted the distributions of essential and nonessential genes versus their morphological signal score (see below for the definition).

### UMAP clustering of the hit perturbation profiles

To evaluate and demonstrate the ability of morphological profiles to uncover biologically relevant interactions and structures, the UMAP algorithm was used to project the hit gene profiles into a two-dimensional plane. PCA was performed on the datasets to capture at least 90% of the variation as described above before the application of the UMAP algorithm. The Python library UMAP was used to apply the UMAP algorithm using ‘cosine’ for parameter ‘metric’. The details of the parameters used are available on the GitHub repository. Some of the resulting clusters were manually labeled to highlight some underlying interesting biology using GO terms (biological processes and cellular components) as listed on the GSEA-MSigDB web portal (http://www.gsea-msigdb.org/gsea/msigdb/human/collections.jsp#C5).

### Hierarchical clustering of hit perturbation profiles and representative heat maps

Correlations between morphological profiles is a powerful tool to extract biological insights from datasets. For example, similarity (or dissimilarity) contains information regarding functional clusters, protein structure, signaling pathways and their directionality. To this purpose, first, PCA was performed on the datasets to capture at least 90% of the variation as described above followed by the selection of a subset of perturbations associated with a functional gene set as specified in each instance. Then, the corr function from the pandas library in Python was used to calculate the pairwise Pearson correlation coefficient of the perturbation profiles for each dataset. The hierarchical clustering of the correlations and the plotting of the heat maps was performed using the seaborn’s clustermap function in Python. The ward variance minimization was used as the clustering algorithm (‘method’) based on the ‘euclidean’ as the distance metric.

For the combined heat maps used in Fig. 3e,f to compare DMEM and HPLM screens, the above process was performed on one screen as explained (with no heat maps generated at this step). Then, the order of clustering was extracted from one screen and applied to the other screen to enable two types of comparisons: direct comparison between correlations from two screens and high-level structural comparison in the clustered correlations. To effectively illustrate the output, both sets of ordered correlations were merged into a single heat map with the bottom left half representing one screen and the top right representing the other, using the seaborn.clustermap^81^ function in Python.

### Preranked GSEA analysis of perturbations based on morphological signal strength or similarity

To better understand the biological processes highlighted in each HeLa screen and to compare the phenotypic downstream effects of the environment on cells, preranked GSEA analysis was performed. The analysis was performed on the GSEA v.4.2.3 Mac software and the genes were ranked based on the morphological signal score using the ‘c5.go.bp.v2022.1.Hs.symbols.gmt [Gene ontology]’ gene set database with 2,000 permutations. The morphological signal score was calculated using this equation for each perturbation

$${\mathrm{morphological}}\;{\mathrm{signal}}\;{\mathrm{score}}=\mathop{\sum }\limits_{i=1}^{n}\left(-\log ({P\;{\mathrm{value}}}_{i})\right).$$

The *P* values were calculated as described in the hit-calling section, and *n* refers to features significantly different from the nontargetting controls (*P* value of 0.001). The code used to calculate the morphological signal score as well as the list of perturbation scores for each dataset is available on the GitHub repository. The EnrichmentMap application based on the Cytoscape v3.9.1 software platform was used to visualize the enrichment maps (node cut-off *q* value of 0.05).

Preranked GSEA analysis was performed to determine enrichment for biological terms based on the morphological profile similarity to a query gene of interest. Genes were ranked based on cosine similarity to the profile of the query gene, then GO term enrichment was performed using the GSEApy package and the ‘GO_Cellular_Component_2021’ database.

### Single feature screen analysis

For each feature in the feature-selected dataset, genes were sorted by *P* value (as generated during hit calling) and a top 20+ list was created for each feature that contained all genes with a *P* value less than or equal to that of the 20th gene. The top 20+ list was assessed for GO term enrichment using the Python GOATOOLS library^82^ with the default Benjamini–Hochberg FDR correction. GO terms were considered enriched if they had a *P* value of <0.05 after an additional Bonferonni correction. Compartment-specific gene lists were assayed for enrichment in the top 20+ lists using a Fisher exact test with a Benjamini–Hochberg FDR correction from the Python SciPy library^83^. Plots were made with Python library Matplotlib^84^. For exploration of granularity features, guide normalized but not feature-selected datasets were aggregated with Pycytominer and plotted with Seaborn^81^. Gene lists were taken from the Metabolic Atlas^85^. Granularity features were visualized with Python SciPy and scikit-image^86^ libraries as implemented in CellProfiler.

### Atlas cell retrieval tool

Example single-cell image crops can be retrieved from any of the screens using a retrieval script included in our paper repository at https://github.com/broadinstitute/2022_PERISCOPE. Images are retrievable by gene name or sgRNA barcode sequence and example images can be chosen randomly or set to the most representative cells for that barcode as determined by closest *k*-means clustering using scikit-learn^87^. Individual channel crops are from corrected images on which the final analysis measurements are made. Mask crops are from segmentations generated during the analysis pipeline and are filled light gray to show the cell of interest and dark gray to show cells within the same crop assigned to the same perturbation.

### TMEM251 localization assay

HT1080 cells were transduced with lentiviral vectors expressing either TagBFP-tagged GALNT2 (Golgi) or mRFP1-tagged TMEM192 (lysosome), and selected with antibiotics. Cells with stable integration were fixed with 4% formaldehyde (15 min at 4 °C), permeabilized with 20 µg ml^−1^ digitonin (30 min at room temperature), blocked with 1% BSA (30 min at room temperature) and incubated with primary antibody against TMEM251 (HPA048559, Sigma-Aldrich; 1:200 overnight at 4 °C) followed by Alexa Fluor 488-conjugated secondary antibody (1:1,000, 2 h at room temperature). Samples were imaged on the Phenix imager (Perkin-Elmer) with a 63× objective in confocal mode.

### WGA/LAMP1 costaining and quantification of lysosomal glycan accumulation followed by CRISPRi perturbations

HT1080 CRISPRi cells were transduced with sgRNA-expressing lentiviral vectors and selected with antibiotics. For dual-target samples, cells were transduced simultaneously with two vectors and coselected with two antibiotics. At 8 days after sgRNA transduction and 2 days after final replating, cells were fixed, permeabilized, blocked and stained as above, using primary antibody against LAMP1 (ab25630, Abcam; 1:50) and Alexa Fluor 647-conjugated secondary antibody. Alexa Fluor 555-conjugated WGA at 1.5 µg ml^−1^ and Hoechst 33342 at 5 µg ml^−1^ were included during secondary antibody incubation. Samples were imaged on the Phenix imager (Perkin-Elmer) with a 63× objective in confocal mode.

Image analysis was performed using the Harmony software (Perkin-Elmer), where images were flat field corrected and regions corresponding to the nucleus, cytoplasm and lysosome were identified. WGA signals that colocalized with the lysosomes were quantified by the median fluorescence intensity (MFI) for each cell. Each biological replicate (two per condition) was represented by the upper quartile of the per-cell MFIs from all segmented cells.

### Lysosomal pH measurement

HT1080 CRISPRi cells stably expressing rat Lamp1 tagged with mScarlet (on the lumen side) were transduced with sgRNA-expressing lentiviral vectors and selected with antibiotics. Cells were imaged live, in an environmental control chamber (OKO) at 37 °C and 5% CO_2_, 8 days after sgRNA transduction and 1 day after replating into imaging media on an 8-well chambered cover glass (Cellvis, C8-1.5H-N). Imaging was performed on an SP8 scanning microscope (Leica) in FLIM mode using a 100× objective. Samples were excited by a white light laser at 561 nm and 40 MHz, and emission collected between 590 and 700 nm. Imaging media consisted of FluoroBrite DMEM (Life Technologies, A1896701) + 10% FBS + 1% GlutaMax (Gibco, 35050061).

Image analysis was performed using in-house scripts, which identified lysosomal regions and the mean arrival time (lifetime) of photons in each pixel. The median lifetime from all lysosomal pixels in each field of view (consisting of one to two cells each, with ≥15 fields per condition) was computed and represented as one data point per field of view. After the initial imaging, 100 nM Bafilomycin A1 was added to the nontargeting sample for a positive control, which was re-imaged 5 h after the treatment.

### Lysosomal hydrolase activity assay

HT1080 CRISPRi cells were transduced with sgRNA-expressing lentiviral vectors and selected with antibiotics. At 9 days after sgRNA transduction and 1 day after final replating, cells were assayed for their lysosomal hydrolase activity by incubating with 0.2 µg ml^−1^ Hoechst 33342 and either 200 µM PFB-FDGlu (for glucosylceramidase; Invitrogen, P11947) or 33 µM C_12_FDG (for beta-galactosidase; Invitrogen, I2904) in imaging media for 1 h at 37 °C, before imaging on the Phenix imager (Perkin-Elmer) with a 63× objective in confocal mode.

Imaging analysis was performed using the Harmony software (Perkin-Elmer), where flat field-corrected images were segmented for nucleus and cytoplasm. Total fluorescence intensity for each cell was extracted, and each biological replicate (two per condition) was represented by the median of the per-cell fluorescence (MFI) from all segmented cells, relative to the nontargeting controls, as log_10_ fold change.

### Reporting summary

Further information on research design is available in the Nature Portfolio Reporting Summary linked to this article.

## Online content

Any methods, additional references, Nature Portfolio reporting summaries, source data, extended data, supplementary information, acknowledgements, peer review information; details of author contributions and competing interests; and statements of data and code availability are available at 10.1038/s41592-024-02537-7.

## Supplementary information

Supplementary information Supplementary Fig. 1. Phenotypic consequences of lysosomal trafficking perturbations (sample images contributing to Fig. 6).

Reporting Summary

Supplementary Tables 1–4 Supplementary Table 1 Barcodes. This table contains all sgRNA sequences and sources, oligo sequences for sgRNA cloning into the CROP-seq vector, and gene IDs and symbols. Supplementary Table 2 Hit lists 1 FDR. This table contains hit genes identified the in whole-cell and compartment-specific categories with their corresponding number of significant features per phenotypic channel. Supplementary Table 3 GSEA clusters. This table contains GSEA clusters and their respective statistics for the HeLa DMEM and HeLa HPLM datasets. Supplementary Table 4 Dilution factors phenotypic probes. This table contains dilution factors of phenotypic probes used in each genome-wide screen.

## Data Availability

All data are publicly available. Phenotyping and ISS images and image-based profiles are available in the Cell Painting Gallery^80^ on the Registry of Open Data on AWS (https://registry.opendata.aws/cellpainting-gallery/) under accession number cpg0021-periscope. Instructions for retrieving images and profiles are available within the Cell Painting Gallery documentation via GitHub at https://github.com/broadinstitute/cellpainting-gallery. Image based profiling data is welded to individual datasets using a template available via GitHub at https://github.com/broadinstitute/pooled-cell-painting-profiling-template. It is processed with a recipe available via GitHub at https://github.com/broadinstitute/pooled-cell-painting-profiling-recipe. The recipe outputs for the datasets that we report here are available via GitHub at https://github.com/broadinstitute/CP186-A549-WG and https://github.com/broadinstitute/CP257-HeLa-WG. The comparison between pairwise correlation of perturbations to other databases was performed using the 28.11.2022 CORUM4.0 database (https://mips.helmholtz-muenchen.de/corum/download) and the STRING v11.5, ‘9606.protein.links.v11.5.txt.gz’ (https://version-11-0.string-db.org/cgi/download.pl?).

## Code availability

All code is publicly available. The recipe outputs were further processed to generate the profiles analyzed in this paper. Code for the final processing and the creation of all figures in this paper are available via GitHub at https://github.com/broadinstitute/2022_PERISCOPE. The exact CellProfiler pipelines used in the screen are available in the Cell Painting Gallery^80^, while continuously improved image analysis pipelines and AWS Lambda scripts used to trigger them are available via GitHub at https://github.com/broadinstitute/pooled-cell-painting-image-processing.
