# Supplementary material for: A genome-wide atlas of human cell morphology
Source: Nat Methods. 2025 Jan 27;22(3):621–33. doi: 10.1038/s41592-024-02537-7 (PMC11903339; doi:10.1038/s41592-024-02537-7)
Supplement: Supplementary file 1 — Supplementary Fig. 1. Phenotypic consequences of lysosomal trafficking perturbations (sample images contributing to Fig. 6). [file 41592_2024_2537_MOESM1_ESM.pdf]

nature methods

Supplementary information https://doi.org/10.1038/s41592-024-02537-7

A genome-wide atlas of human cell morphology

In the format provided by the authors and unedited

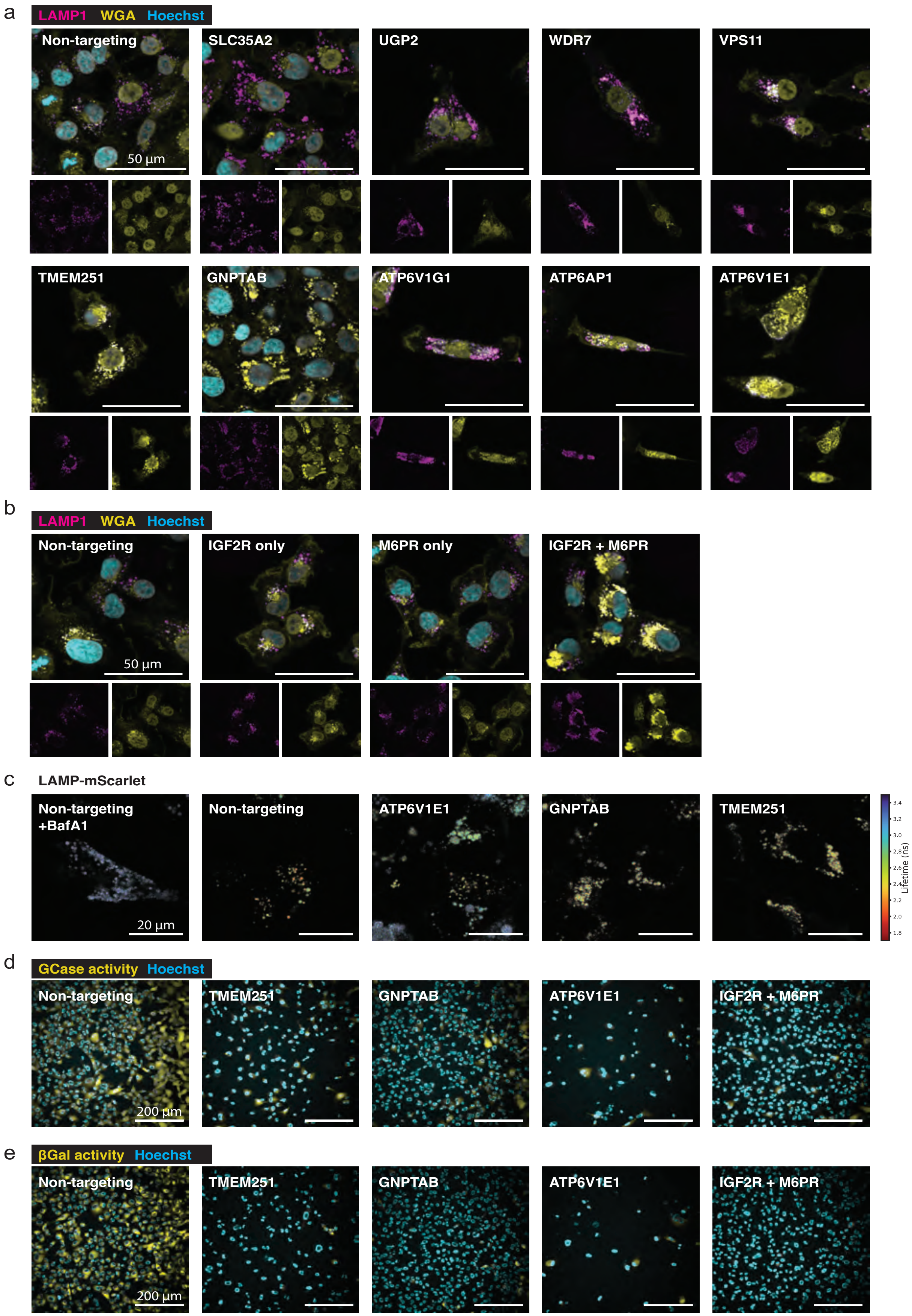

Supplementary Figure 1. Phenotypic consequences of lysosomal trafficking perturbations (sample images contributing to Figure 6)

(a) WGA and LAMP1 co-staining of cells with knockdown (KD) genes highlighted in Figure 6b. Quantification of the imaging data is shown in Figure 6e. (b) WGA and LAMP1 co-staining of cells with single or dual gene KD as indicated. Quantification of the imaging data is shown in Figure 6f. (c) Color overlays of mScarlet-Lamp1 cells with KD of the genes indicated. Image intensity represents photon count per pixel, whereas hue encodes median lifetime per pixel. The images shown are representative of patterns observed in n=30 images for GNPTAB and TMEM251; n=15 images for the remaining conditions. Quantification of the imaging data is shown in Figure 6g. (d-e) Live cells with KD of the genes indicated and incubated with fluorogenic substrates of glucosylceramidase (d) and beta-galactosidase (e), respectively. Quantified data are shown in Figure 6h-i. The confocal images shown in (a), (b), (d), & (e), are representative of the staining patterns observed in two biological replicates per gene target.
