# Supplementary material for: A genome-wide atlas of human cell morphology
Source: Nat Methods. 2025 Jan 27;22(3):621–33. doi: 10.1038/s41592-024-02537-7 (PMC11903339; doi:10.1038/s41592-024-02537-7)
Supplement: Supplementary file 2 — Reporting Summary [file 41592_2024_2537_MOESM2_ESM.pdf]

nature portfolio

Corresponding author(s): James T. Neal

Last updated by author(s): October 4th, 2024

Reporting Summary

Nature Portfolio wishes to improve the reproducibility of the work that we publish. This form provides structure for consistency and transparency in reporting. For further information on Nature Portfolio policies, see our Editorial Policies and the Editorial Policy Checklist.

Statistics

For all statistical analyses, confirm that the following items are present in the figure legend, table legend, main text, or Methods section.

n/a	Confirmed	
☐	☒	The exact sample size (*n*) for each experimental group/condition, given as a discrete number and unit of measurement
☐	☒	A statement on whether measurements were taken from distinct samples or whether the same sample was measured repeatedly
☐	☒	The statistical test(s) used AND whether they are one- or two-sided<br>*Only common tests should be described solely by name; describe more complex techniques in the Methods section.*
☒	☐	A description of all covariates tested
☐	☒	A description of any assumptions or corrections, such as tests of normality and adjustment for multiple comparisons
☐	☒	A full description of the statistical parameters including central tendency (e.g. means) or other basic estimates (e.g. regression coefficient) AND variation (e.g. standard deviation) or associated estimates of uncertainty (e.g. confidence intervals)
☐	☒	For null hypothesis testing, the test statistic (e.g. *F*, *t*, *r*) with confidence intervals, effect sizes, degrees of freedom and *P* value noted<br>*Give P values as exact values whenever suitable.*
☒	☐	For Bayesian analysis, information on the choice of priors and Markov chain Monte Carlo settings
☒	☐	For hierarchical and complex designs, identification of the appropriate level for tests and full reporting of outcomes
☐	☒	Estimates of effect sizes (e.g. Cohen's *d*, Pearson's *r*), indicating how they were calculated

Our web collection on statistics for biologists contains articles on many of the points above.

Software and code

Policy information about availability of computer code

Data collection	Phenotypic and 155 images were acquired using a Nikon Ti-2 Eclipse inverted epifluorescence microscope, and included Nikon NIS Elements AR software (version 5.42.01).
Data analysis	We used Cell Profiler bioimage analysis software (version 4.1.3) to process the images using classical algorithms and Fiji (with openjdk-8) for image stitching and cropping. We processed outputs of Cell Profiler into image-based profiles using scripts available at https://github.com/broadinstitute/pooled-cell-painting-profiling-recipe. Each dataset is independently welded to the recipe, effectively versioning the recipe, using a Template, available at https://github.com/broadinstitute/pooled-cell-painting-profiling-template. Code used for further profile processing is in this paper repository at https:// github.com/broadinstitute/2022_PERISCOPE. The EnrichmentMap application based on the Cytoscape v3.9.1 software platform was used to visualize the enrichment maps (node cutoff Q-value 0.05).

For manuscripts utilizing custom algorithms or software that are central to the research but not yet described in published literature, software must be made available to editors and reviewers. We strongly encourage code deposition in a community repository (e.g. GitHub). See the Nature Portfolio guidelines for submitting code & software for further information.

Data

Policy information about availability of data

All manuscripts must include a data availability statement. This statement should provide the following information, where applicable:

- Accession codes, unique identifiers, or web links for publicly available datasets
- A description of any restrictions on data availability
- For clinical datasets or third party data, please ensure that the statement adheres to our policy

All code and data are publicly available. Phenotyping and in situ sequencing images and image-based profiles are available at the Cell Painting Gallery on the Registry of Open Data on AWS (https://registry.opendata.aws/cellpainting-gallery/) under accession number cpg0021-periscope. Instructions for retrieving images and profiles are available within the Cell Painting Gallery documentation at https://github.com/broadinstitute/cellpainting-gallery. Image based profiling data is welded to individual datasets using a template available at https://github.com/broadinstitute/pooled-cell-painting-profiling-template. It is processed with a recipe available at https://github.com/broadinstitute/ pooled-cell-painting-profiling-recipe. The recipe outputs for the datasets that we report here are available at https://github.com/broadinstitute/CP186-A549-WG and https://github.com/broadinstitute/CP257-Hela-WG. Comparison between pair-wise correlation of perturbations to other databases was performed using the 28.11.2022 CORUM4.0 database (https://mips.helmholtz-muenchen.de/corum/download) and the STRING v11.5, "9606.protein.links.v11.5.txt.gz" (https://version-11-0.string-db.org/cgi/download.pl?).

Human research participants

Policy information about studies involving human research participants and Sex and Gender in Research.

Reporting on sex and gender	N/A
Population characteristics	N/A
Recruitment	N/A
Ethics oversight	N/A

Note that full information on the approval of the study protocol must also be provided in the manuscript.

Field-specific reporting

Please select the one below that is the best fit for your research. If you are not sure, read the appropriate sections before making your selection.

☒ Life sciences ☐ Behavioural & social sciences ☐ Ecological, evolutionary & environmental sciences

For a reference copy of the document with all sections, see nature.com/documents/nr-reporting-summary-flat.pdf

Life sciences study design

All studies must disclose on these points even when the disclosure is negative.

Sample size	No sample size calculation was performed, we screened as many cells as we could fit in 6-8 6-well plates. This number of plates was determined to be the largest number we could screen concurrently with available personnel and equipment.
Data exclusions	No data were excluded from analysis, except cells that were filtered out because we couldn't assign them a perturbation barcode.
Replication	All screens were executed in three independent biological replicates, and all attempts at replication were successful.
Randomization	Not applicable. Control and non-control perturbations are measured simultaneously at genome-scale and analyzed in automated fashion.
Blinding	Not applicable - Data analysis was performed in an automated manner using pipelines described in the paper.

Reporting for specific materials, systems and methods

We require information from authors about some types of materials, experimental systems and methods used in many studies. Here, indicate whether each material, system or method listed is relevant to your study. If you are not sure if a list item applies to your research, read the appropriate section before selecting a response.

Materials & experimental systems

n/a	Involved in the study
☐	☒ Antibodies
☐	☒ Eukaryotic cell lines
☒	☐ Palaeontology and archaeology
☒	☐ Animals and other organisms
☒	☐ Clinical data
☒	☐ Dual use research of concern

Methods

n/a	Involved in the study
☒	☐ ChIP-seq
☒	☐ Flow cytometry
☒	☐ MRI-based neuroimaging

Antibodies

Antibodies used	Anti-LAMPl antibody [H4A3] (ab2S630), abeam. Anti-TOMM20 antibody - Mitochondrial Marker (ab78547), abcam. Anti-TMEM251 antibody (HPA048559, Sigma-Aldrich)
Validation	anti-lampl validation provided by abcam: ICC, IHC, and Western blot. anti-TOMM20 validation provided by abeam: ICC, IHC, and Western blot. anti-LAMPI was additionally checked for specificity against another LAMPI primary antibody (CST), and anti-TOMM20 was checked against MitoTracker Deep Red. anti-TMEM251 validation provided by Sigma-Aldrich: ICC, IHC, and Western blot.

Eukaryotic cell lines

Policy information about cell lines and Sex and Gender in Research

Cell line source(s)	HT1080: ATCC. A549 (male lung cancer): ATCC. Hela (female cervical cancer): lain Cheeseman (MIT/Whitehead), parental line sourced from ATCC. HEK293FT: Thermo Fisher Scientific.
Authentication	All cell lines were authenticated using ATCC's human STR profiling service
Mycoplasma contamination	All cell lines tested negative for mycoplasma contamination.
Commonly misidentified lines (See ICLAC register)	No commonly misidentified lines were used in this study.
